# Supplementary material for: Growth of magnetic nanowires along freely selectable 〈hkl〉 crystal directions
Source: Nat Commun. 2018 Jan 23;9:339. doi: 10.1038/s41467-017-02519-8 (PMC5780464; doi:10.1038/s41467-017-02519-8)
Supplement: Supplementary file 1 — Supplementary Information [file 41467_2017_2519_MOESM1_ESM.pdf]

Supplementary Note 1

1. The Origin of Deposition Rate Dependence

During homoepitaxial nanowire elongation on biaxial, single-crystalline seeds, newly deposited adatoms on the growing tips possess sufficient mobility to diffuse and to sample the low-energy sites, before they are immobilized in crystal lattice sites [1]. The observation that lower evaporation rates (0.25 nm/sec: Supplementary Figure 1a) do not lead to single-crystalline growth suggests that the growth conditions at higher rates (3.0 nm/sec: Supplementary Figure 1b) impart both sufficient mobility to the adatoms, and a suitable (e.g.: clean) growth-front crystal surface on which the atoms can freely diffuse and crystallize. The purpose of this section is to identify the likely origin of this rate dependence (Supplementary Figure 1). The discussion is based on calculations and control experiments. A more detailed mechanistic understanding or hypotheses would help guide future work.

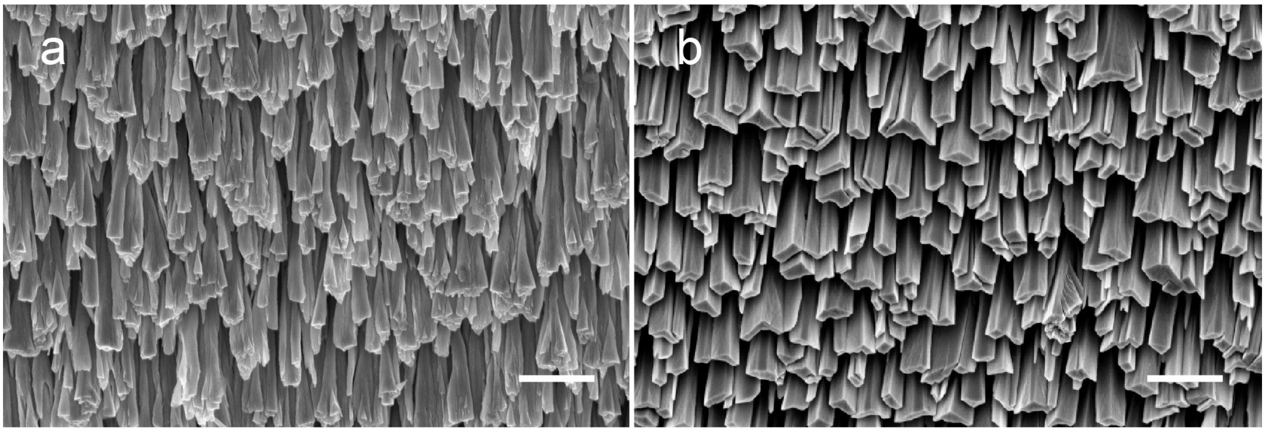

Supplementary Figure 1: Single-crystalline growth requires sufficient deposition rate. a Sample grown at 0.25 nm/sec. **b** Sample grown at 3 nm/sec. Scale bars are 1 μm

Two of the most likely mechanistic bases are the following:

A. Faster deposition causes higher temperature at the tip of the nanowires, which in turn increases the mobility of the adatoms to facility homoepitaxy at the growth front [2–4].

B. Faster deposition conditions effectively dilute the concentrations of residual gas species in the deposition chamber, which can hinder homoepitaxy by their adsorption onto the growth surface [5–9].

These mechanisms can operate both alone or in concert. To test if thermal energy plays an important role, we estimate the temperature gradient along the nanowires by measurements and calculations. We measure the heating power during deposition, calculate the thermal resistance of the nanowires

and the interface resistance with the substrate, and derive the range of possible temperature difference between substrate and nanowire tip. The results allow us to tentatively rule out deposition-induced heating as a major contributor to the experimental observation. We therefore propose residual gas effects as the more likely cause. This conclusion has important implications for future development and generalization of the technology for arbitrary crystallinity control by GLAD demonstrated in the present work.

This section is organized as follows:

In Subsection 1.1., we estimate the temperature of the FeCo source as a function of deposition rate to enable a more accurate calculation of the heating power by vapor condensation for different deposition rates.

In Subsection 1.2., we present and discuss control experiments that semi-quantitatively measure the total power deposited on the growing nanowires during their growth. We show that photon irradiation can also deposit significant thermal energy onto the substrate, comparable to or even more than does vapor condensation.

In Subsections 1.3.-1.4., we estimate by calculation the thermal conductance of the nanowires to determine whether they are able to sustain a significant temperature gradient across their length. We find that, even when nanostructuring were taken into account, the expected thermal conductance for the naowires is too high to maintain any appreciable temperature gradient, so cannot explain the observed effect.

In Subsection 1.5., we discuss the possible existence of significant interface thermal resistance between the bottom end of the nanowires and the substrate that would allow for a sufficient temperature difference between the substrate and the nanowires.

In Subsection 1.6., we discuss the elementary mechanisms important to single-crystalline epitaxy and calculate the 2D diffusion lengths the adatoms can exhibit as a function of deposition rate and substrate temperature. We conclude that the deposition rate must somehow affect the diffusion behavior through other mechanisms.

Finally, in Subsection 1.7., we offer alternative explanations for the observed rate dependence of single-crystal growth. We also suggest future experiments that could be be conducted to test these ideas.

1.1. Temperature of vapor source as a function of deposition rate

The rate of thinfilm growth by ebeam evaporation as a function of material properties, chamber geometric conditions, and source temperature can be derived from the Hertz-Knudsen equation [10],

which gives the rate of material departure, Γ (kg/sec), from a heated source with surface area A_e (m^2) as

$$\Gamma = A_e(\frac{m}{2\pi k_B T})^{1/2}[P(T) - P_0] \tag{1}$$

where m is the mass (kg) of a single evaporant atom or molecule, k_B is Boltzmann's constant, T is the temperature (K), $P(T)$ is the vapor pressure (pascal) of the evaporant at T, and P_0 is the background vapor pressure (pascal) of the evaporant. Under ebeam evaporation conditions, P_0 is negligibly small and can be dropped (i.e. that there is very little chance for evaporated material to return back into the crucible.), giving

$$\Gamma = A_e(\frac{m}{2\pi k_B T})^{1/2} P(T) \tag{2}$$

It is a well-known result that if the source were placed on the inner surface of a hollow sphere, facing inward, the spherical shell's inner surface would be coated with a uniform thickness of the evaporated material [10]. The total inner surface area of a spherical shell with inner radius $r = D/2$ (D is the source-sample distance) is $4\pi r^2 = \pi D^2$. Therefore, the *areal* rate of material deposition (kg sec^{-1} m^{-2}) on a sample surface, placed directly above the source, is

$$\Gamma_{\text{areal}} = A_e(\frac{m}{2\pi^3 k_B T})^{1/2} \frac{P(T)}{D^2} \tag{3}$$

For subsequent calculations, it is easier to use molar mass ($N_a m \equiv M$) of the evaporant rather than to use the kg mass of single atoms by invoking Avogadro's number, N_a. Using $R = N_a k_B$, we have

$$\Gamma_{\text{areal}} = A_e(\frac{1}{2\pi^3 R})^{1/2} (\frac{M}{T})^{1/2} \frac{P(T)}{D^2} \tag{4}$$

The goal of this calculation is to relate source temperature to a deposition rate, dh/dt, in dimensions of length over time. This is achieved by dividing Eqn. 4 by the density of the evaporant, ρ (kg/m^3).

$$\frac{dh}{dt} = \frac{\Gamma_{\text{areal}}}{\rho} = \frac{A_e}{\rho}(\frac{1}{2\pi^3 R})^{1/2} (\frac{M}{T})^{1/2} \frac{P(T)}{D^2} \tag{5}$$

Using Eqn. 5 and literature data for the vapor pressures of pure iron and cobalt [11], we plot the temperature of the source, assumed to have an area of 1 cm^2 (Supplementary Figure 11b), as a function of measured deposition rate (Supplementary Figure 2). The results graphically show that for pure Fe and pure Co, when the evaporation increases from 0.25 nm/sec to 3.0 nm, the source temperature changes by about 200-300 degrees. Even though the alloy is expected to show a vapor

pressure that is intermediate between the constituent elements, the trend will be the same. Therefore, the vapor at higher evaporation rate has at most $\frac{3}{2}R\Delta T = 3.7\text{kJ/mol}$ more energy than the vapor at the lower evaporation rate. The remaining parts of this section are devoted to discussing if and how this extra energy contained in the hotter vapor could lead to the experimentally observed difference (Supplementary Figure 1). It will be shown that the contribution of a few kJ/mol is negligible and cannot explain the experimental observation.

1.2. Sources of thermal power: material flux vs photon flux

Thermal energy deposits onto the sample during ebeam evaporation via two dominant mechanisms [12, 13]:

- condensation and cooling of material vapor
- photon irradiation from a glowing molten source

To obtain a sense of the total magnitude and the relative contributions of the heating powers of these two mechanism under the experimental conditions, we recorded the temperature of large (152mm × 114mm × 0.25mm) borosilicate glass ($\kappa = 1.2$ W m^{-1} K^{-1}; $C_P = 1.66$ J cm^{-3} K^{-1}) substrates (Kurt J. Lesker) during the evaporation of FeCo for the lowest and highest evaporation rates used in this study. The substrate geometry and material were chosen to increase the thermal time constant of the nanowire-substrate system, thus minimizing the loss of heat into the sample holder. To minimize heat transfer, care was taken to restrict the contact area between the substrate and sample holder to about 1 cm^2, at a point several centimeters away from the point of temperature measurement.

A metal thermocouple was used for the measurement and was fixed to the backside of the glass by a drop of silver paint. The drop of silver paint dried to roughly 5mm × 5mm in area and 1 mm in

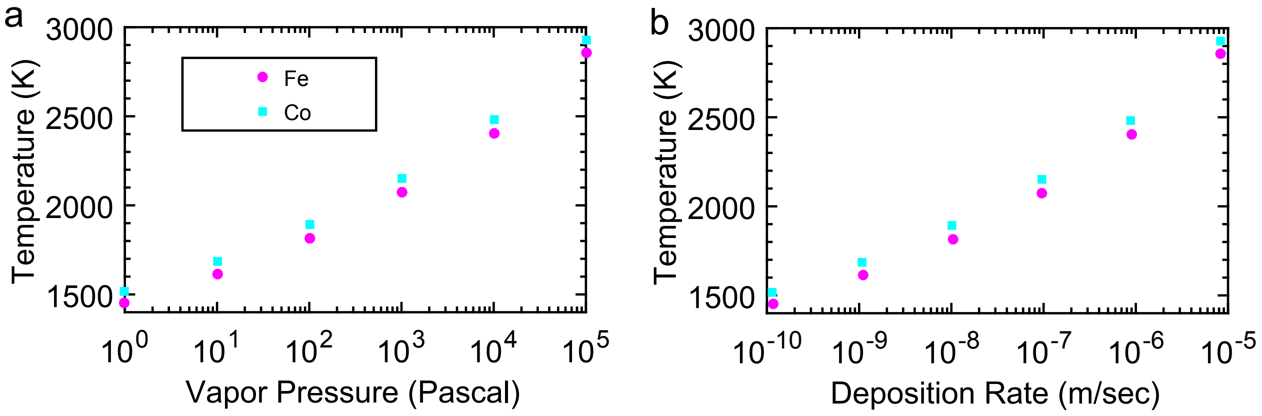

Supplementary Figure 2: Source Temperature vs Deposition Rate. **a** Temperatures of pure Fe and Co as a function of their vapor pressures [11]. **b** Temperatures of pure Fe and Co sources as a function deposition rate, calculated based on Eqn. 5.

height. Assuming the dried paint had the heat capacity of silver (2.24 J cm^{-3} K^{-1}), the painted area would have a areal heat capacity that is about 6 times that of the glass with the same area. This is a conservative overestimation for two reasons. First, the dried paint will have a density that is lower than that of pure silver. Second, during FeCo evaporation and measurement, some heat from the glass substrate in the surrounding areas will flow toward the dried silver paint, lowering the contribution of the paint to the effective areal heat capacity. Therefore, the effective areal heat capacity of the glass-silver paint measurement spot falls between 1-7 times that of the glass substrate. Since the latter can be accurately calculated, we expect the following analysis and calculation to give an estimate that is accurate to well within an order of magnitude.

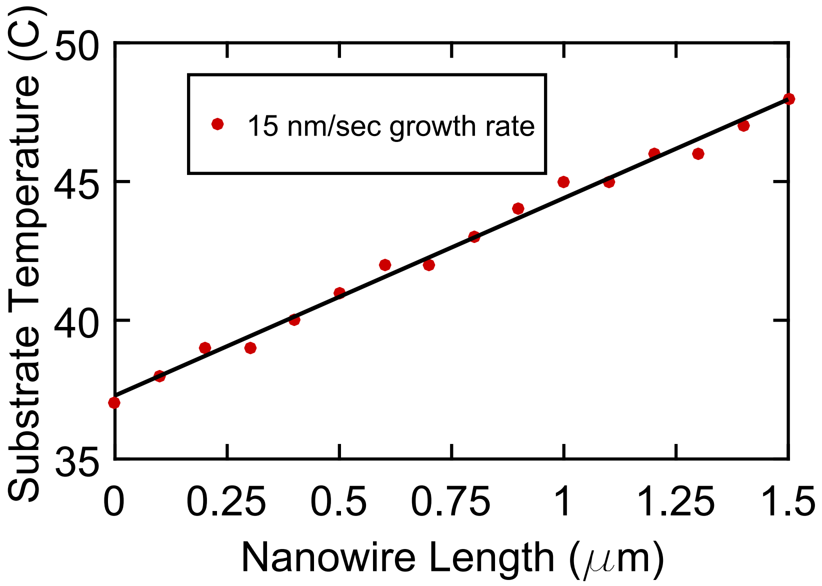

Supplementary Figure 3: Heating of substrate during evaporation. Data were recorded using thermocouple on the backside of a large borosilicate glass slide used as substrate. See text for detailed discussion.

Supplementary Figure 3 shows the change in measured substrate temperature as a function of the length of grown nanowires, at a constant nanowire growth rate of 15 nm/sec. The data covers a period of 100 seconds. The linearity in the data suggests that little heat is lost by conduction on this time scale so that the heating power can be extracted. A linear fit to the data shows that the substrate heats up with a slope of 7.1(4) K/μm of nanowire growth. We need to convert this number to power per exposed area normal to vapor flux, which is smaller than the sample area by a factor $\cos\alpha$. So the heating rate per unit length deposition of a dense film perpendicular to flux is $7.1(4)/\cos 85 = 81.(4)$ K μm^{-1} = 8.1(4) $\times 10^5$ K cm^{-1} FeCo deposition. To convert this number to energy density per deposited FeCo (note that this is rate-dependent due to a varying contribution from radiation heating), we use the heat capacity of borosilicate glass (1.66 J cm^{-3} K^{-1}), the thickness of the substrate (250 μm), and the fact that the glass-silver paint system can

have up to 7 times the heat capacity of the glass alone. The lower bound for the thermal energy deposited per volume of FeCo deposition is therefore

$$(8.1 \times 10^5 \frac{\text{K}}{\text{cm}})(1.66 \frac{\text{J}}{\text{cm}^3\ \text{K}})(0.025\text{cm}) = 3.4 \times 10^4 \frac{\text{J}}{\text{cm}^3} \tag{6}$$

The molar volume of FeCo alloy is 7.27 cm^3. Therefore, the total thermal energy deposited on the substrate per mole of arriving FeCo at a rate of 15nm/sec is between 247 kJ and 7 times that at 1730 kJ. This range of values can be compared to the expected heat released upon condensation of a FeCo vapor and its cooling to near room temperature. From Supplementary Figure 2b, we estimate the vapor at 3 nm/sec deposition rate to be about 300K above the melting point of about 1500K. The cooling of the gas from 1800K to 1500K would therefore release $\frac{3}{2}R\Delta T = 3.7\text{kJ/mol}$. The heat of vaporization of $Fe_{0.65}Co_{0.35}$ is two orders of magnitude higher at 357 kJ/mol. We therefore see that any temperature difference in the source (Supplementary Figure 2) contributes very little to the molar heating energy at the substrate by material flux. The heat of fusion is about 16 kJ/mol. And the heat capacity of FeCo is about 25 J mol^{-1} K^{-1}. When cooling from a melting point of about 1500 K to room temperature, this would give about 30 kJ/mol. As a result, we expect material flux alone to provide about 408 kJ/mol. This number is within the experimentally estimated range of total heating power (247-1730 kJ/mol). The result shows that thermal energy deposited by photon radiation, if present, can be at most 3 times that by material flux at an evaporation rate of 15 nm/sec.

However, photon radiation heating is certainly also present. We found that even though evaporation at low rate leads to a slower rate of substrate heating (Supplementary Figure 4a), the overall heating for the same amount of deposited material is actually higher at a lower evaporation rate (Supplementary Figure 4b). This observation can only result from the existence of significant radiation heating, which is proportionally more significant at lower evaporation rates. Otherwise, the faster run would have led to an higher overall temperature increase.

The above calculations and measurements unequivocally show that both material flux and photon flux contribute to providing thermal energy to the sample and lead to very modest substrate heating by one or two tens of degrees. This conclusion is in line with literature reports for the ebeam evaporation of copper, where photon flux was found to contribute on the order of 10% to the overall substrate heating [13]. The higher contribution of photon heating we observed might be explained by the scaling of black body radiation with T^4 in the Stefan-Boltzmann law. By the melting points of copper and iron, the law would predict that just-molten iron should radiate about 3-4 times more than just-molten copper, whereas their heats of vaporization are within 20% of each other. Additional reasons include the fact that the mechanistic experiment in [13] took care to minimize the photo-

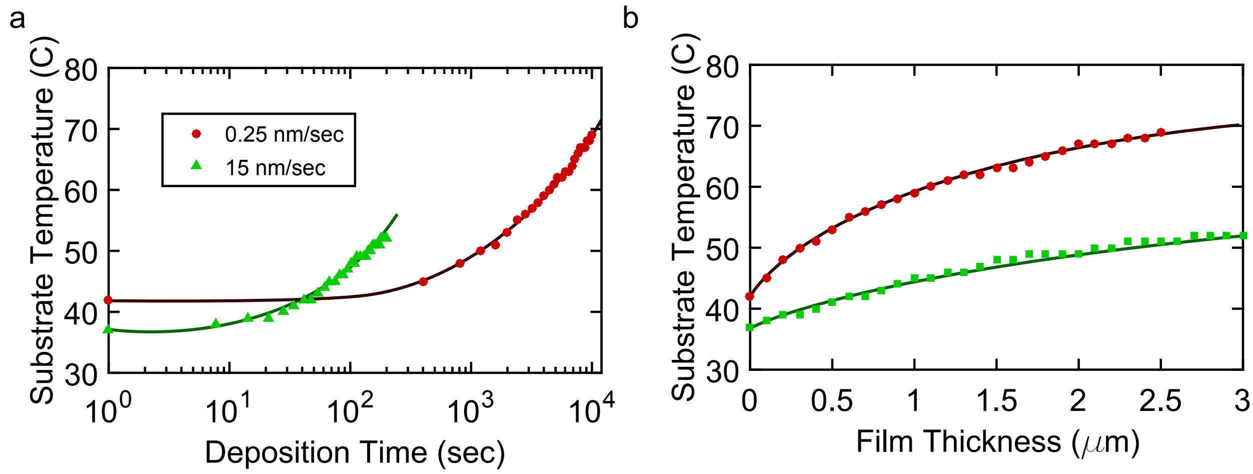

Supplementary Figure 4: Substrate heating due to deposition. Data for the two rate extrema used in the current study are plotted. Solid lines are guides to the eye.

irradiation of the sample by non-evaporating areas of the heated source, whereas our synthetic setup incorporates no such precautions.

1.3. Thermal conductance of nanowires

Having ascertained that there exist mechanisms to provide thermal energy to surface adatoms, and having obtained estimates of the maximum heating power, a next question we wish to ask is the following: What is the temperature difference that can be maintained between the tip and the base of the nanowire? Answering this questions requires a calculation of the nanowires' thermal conductance. This is done in this subsection. Here, we only derive expressions for the thermal conductance as a function of material properties and geometric parameters of the nanowires. We reserve the presentation of numerical results to Subsection 1.4..

We perform the calculations for two nanowire model geometries, straight and tapered, that together capture the range of experimentally observed shapes. We adopt square cross sections for both models to facilitate calculations. We note that as long as the aspect ratio of the cross section were not too extreme, the exact shape of the cross section is inconsequential. The straight geometry would have uniform cross-sectional area of $a \times a$, where a is in the low hundreds of nanometers. The tapered geometry is assumed to start from the substrate making a $a_0 \times a_0$ footprint contact area with the substrate, and terminate with a square tip with area $a \times a$. Based on experiment, $a_0 \leq a$ and is in the range of a few to tens of nanometers. The tapered model fans out conically with apex angle γ, such that $\tan\gamma = \dfrac{a - a_0}{x}$, where x is the length of the nanowire.

Since the length of the nanowires changes (increases) during growth, so does their thermal resistance. It is therefore expected that the temperature difference between the tip and the base increases during the growth. It thus makes sense to write down expressions as a function of nanowire growth. We

work in a coordinate system where the nanowire axis lies on the x axis with one end of the straight nanowire model at the origin. We take the tapered model nanowire to also lie along x, but with the *point of intersection of the extensions of its sides* to coincide with the origin. Thus, its starting end would be located at location x_0, such that $x_0 = \frac{a_0}{\tan\gamma}$.

For a straight nanowire,

$$R_{\text{straight}}(x) = \frac{1}{G_{\text{straight}}(x)} = \frac{x}{\kappa a^2} \tag{7}$$

For a tapered nanowire, the cross-sectional area grows with x as $A(x) = (x\tan\gamma)^2$. The thermal resistance of an infinitesimal slice with thickness dx' is therefore $dR_{\text{tapered}} = \frac{dx'}{\kappa(x'\tan\gamma)^2}$ and

$$R_{\text{tapered}}(x) = \frac{1}{G_{\text{tapered}}(x)} = \int_{x_0}^{x_0+x} \frac{dx'}{\kappa\tan^2\gamma x'^2} \tag{8}$$

$$= \frac{1}{\kappa\tan^2\gamma}\left(\frac{1}{x_0} - \frac{1}{x_0+x}\right) \tag{9}$$

It can be verified that Eqn. 9 reduces to Eqn. 7 by taking the appropriate limits.

1.4. Temperature difference between the ends of growing nanowires

The heating power (Q_{tip}) at the tip of a growing nanowire can be estimated based on the results from Section 1.2.. The thermal resistance (R_{NW}) for representative nanowire geometries were derived in Section 1.3.. One can thus calculate the temperature difference, $\Delta T_{\text{tip-base}} = Q_{\text{tip}}R_{\text{NW}}$, that can be maintained between the growth front at the tip and the bottom end anchored to the substrate.

In the calculations, we take 50 ± 20 (W m^{-1} K^{-1}) as the thermal conductivity of bulk FeCo alloys at temperature ranges above room temperature [14]. We note that transport properties, such as thermal conductivity, are notably geometry-dependent, and have been measured to decrease by a factor of about 3 in metallic nanowires of different materials with geometries and sizes comparable to the FeCo nanowires produced in this study [15–18]. Assuming a reduction by a factor of 3 due to nanostructuring, we use a value of 17 ± 7 (W m^{-1} K^{-1}) as the thermal conductivity of FeCo nanowires. To be more conservative (to thus overestimate $\Delta T_{\text{tip-base}}$), we take the value to be 10 (W m^{-1} K^{-1}) in calculating the results presented in Supplementary Figure 5.

The power at the tip of a single nanowire is equal to the energy density per mole of deposited material multiplied by the number of mole of material deposited per second on the tip. Note that the latter quantity is a constant for the nanowire model with uniform cross sectional area, but increases with length for the tapered nanowire model (Section 1.3.). Results plotted in Supplementary Figure 5 assume that the heating power is the highest possible upper bound based on the measurement

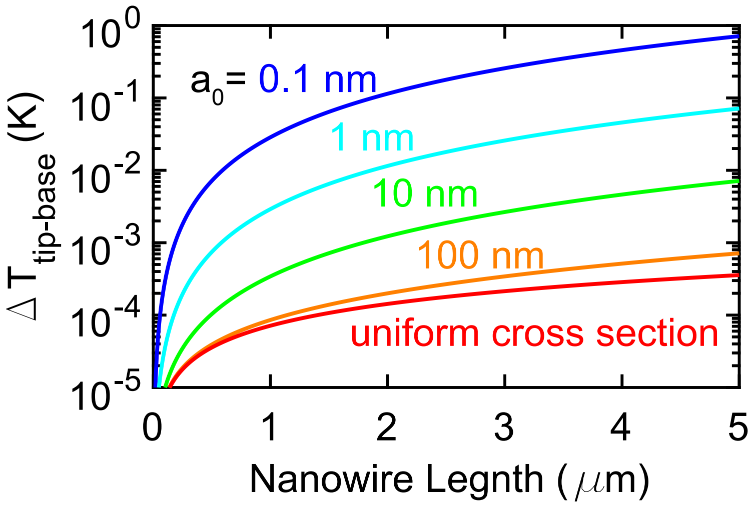

Supplementary Figure 5: Temperature difference between the tip and the base of growing FeCo nanowires as a function of their instantaneous length. The nanowire geometry model with uniform cross-sectional area shows the least temperature change. Tapered nanowire geometry model show increasing temperature gradient as the size of the foot (assume to be square with area $a_0 \times a_0$) shrinks toward the atomic size ($\sim$ 0.1 nm). The cross sectional area at the tip is taken to be $a \times a$ = 300nm$\times$300nm for these calculations. The range of calculated geometries encompasses the range of experimental shapes.

presented in Section 1.2. (1730 kJ/mol).

The uniform-cross section nanowire model shows linear increase in $\Delta T_{\text{tip-base}}$ (not apparent on log scale) because thermal resistance increases linearly with length. The tapered nanowire model shows greater-than-linear increase in $\Delta T_{\text{tip-base}}$ with growth because the heating power increases with length as the nanowire tip widens. The upper-bound estimates plotted in Supplementary Figure 5 shows that the nanowires cannot sustain any appreciable temperature gradient within themselves. In the next subsection, we discuss if a sharp temperature gradient could exist at the boundary with the substrate.

1.5. Nanowire-substrate interface thermal resistance

The interfacial thermal conductance of a range of material combinations have been measured [19]. Existing experimental data and theoretical modeling show that the range of interfacial thermal conductivity, for any combination of two materials, is in a narrow range between 10^7 to 10^9 W m^{-2} K^{-1}.

To obtain an upper bound for $\Delta T_{\text{interface}}$, we use the lower-bound value of 10^7 W m^{-2} K^{-1}. We assume that the contact area is as small as 1nm$\times$1nm. We further maximize heating power by assuming that the nanowire has a top surface area of 300nm$\times$300nm, which would give a maximum heating power of 50 pW at the tip. We therefore have $\Delta T_{\text{interface}} = \dfrac{Q}{G} = \dfrac{50 \times 10^{-12}(\text{W})}{10^7(\text{W m}^{-2}\text{K}^{-1})[10^{-9}(\text{m})]^2} = 5\text{K}$. This upper bound estimate, in the presence of significant background DC temperature fluctuations,

is too small to significantly impact surface mobility. Therefore, it is very likely that the tips of the growing FeCo nanowires are at a temperature that is within a couple of degrees of the substrate temperature throughout the growth process. We conclude that a temperature difference caused by a deposition rate difference is not a likely explanation of the experimental difference (Supplementary Figure 1).

1.6. Adatom diffusion at several temperatures

Results from the preceding sections suggest that the temperature of the nanowire tips are not significantly affected by the deposition rate. Therefore, factors other than adatom mobility induced by deposition heating are likely present. In the following, we give further support for this conclusion by considering the mean diffusion length of adatoms as a function of deposition rate.

We first recall some known results and mechanisms of metal homoepitaxy. Film growth has traditionally been classified into three ideal growth modes [4]:

A. continuous 2D layer-by-layer or Frank-van der Merwe growth (FM)

B. 2D layer-by-layer followed by 3D island formation or Stranski-Krastanov growth (SK).

C. 3D island formation or Vollmer-Weber growth (VW).

To obtain the smooth tip surfaces observed in this study, adatoms on the growth front essentially follows, at least locally, the FM mode, where layers of atoms are laid down one-by-one. This mode of growth is facilitated by several elementary mechanisms, including thermally-activated surface diffusion [3], downward funneling [2, 20], and transient mobility [21]. The latter mechanism, in which incoming atoms keep part of its kinetic energy for an extended period of time due to inefficieintly energy dissipation into the bulk, is highly intuitive and seemingly suggested by experiment [22, 23] for molecular adsorption. It has, however not been experimentally and conclusively demonstrated to exist experimentally for metals. In fact, computational investigations for vapor incidence normal to the surface tend to show that it is negligible for metals [24]. In the FeCo GLAD case, since the incoming vapor is not perfectly perpendicular to the (1 0 0) face, a component of the momentum is parallel to the surface to increase the chance for transient mobility to play a role.

Regardless of whether such more elaborate elementary mechanisms are present, thermally-activivated 2D surface diffusion is certainly the most likely to be important in imparting crystalline order. Surface diffusion constant has the following temperature dependence:

$$D(T) = D_0 \exp(-\frac{E_D}{k_B T}) \tag{10}$$

where $D(T)$ is the diffusion constant, the prefactor D_0 and the activation energy E_d are measured to be 7.2×10^{-4} cm^2 sec^{-1} and 0.45 ± 0.04 eV for the diffusion of Fe on Fe(1 0 0) [3] (Supplementary Figure 6a). Since there have been no measurement of these parameters for the FeCo alloy, we will use the Fe data to get a qualitative sense for the trend in the alloy.

The mean diffusion length in 2D scales with available diffusion time, Δt, as $\sigma_{2D} = \sqrt{4D\Delta t}$. In the case of surface diffusion during physical deposition, Δt is the time an adatom has available before it is captured by some potential well on the surface, or gets immobilized/buried by other incoming atoms. The time needed to deposit one monolayer of the bcc (100) surface (0.145 nm/rate) is a good estimate of the magnitude of Δt. Therefore, the diffusion time available are between 10 msec to 580 msec for deposition rates of 15 nm/sec and 0.25 nm/sec, respectively. We plot the thermal diffusion lengths of adatoms for 3 temperatures of 250K, 300K, and 350K that more than encompasses the range of experimentally measured temperatures as functions of the deposition rate (Supplementary Figure 6b).

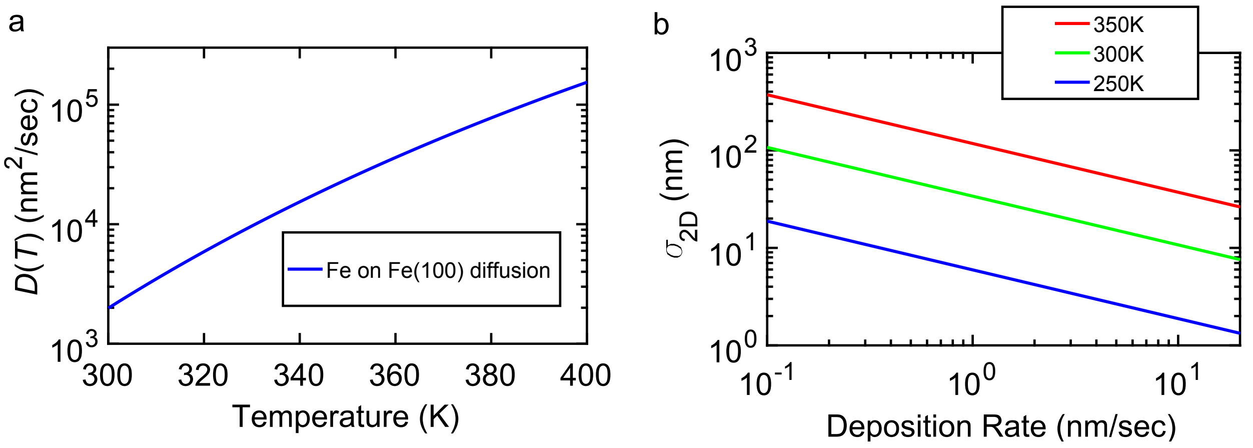

Supplementary Figure 6: Diffusion of Fe adatom on Fe(100) surface. **a** Diffusion constant as a function of temperature. **b** Mean displacement (σ_{2D}) of an Fe adatom during the time need for the deposition of one monolayer of Fe(100) bcc crystal as a function of the deposition rate for 3 temperatures.

The results tell us two things. First, at the deposition temperature used in this study, Fe adatoms have sufficient thermal energy to diffuse for several to tens of nanometers on the time scale of the deposition. Second, more importantly, the diffusion length is longer at slower rate. Intuitively, it is also clear that at lower deposition rates, adatoms have more time to move on the top surface before they are immobilized as the layer becomes completed or as they are buried underneath other incoming atoms. Therefore, slower deposition rate should enable better epitaxy and single crystalline growth with less defects. The experiment is clearly at odds with both of these quantitative and intuitive expectations.

Even when other mechanisms such as downward funneling [20] or transient kinetic mobility were

present [24], one would still expect slower rate to be more beneficial for epitaxy. This is because the kinetic energies of each incoming atom is essentially the same regardless of their total flux, so that the effectiveness of downward funneling and transient kinetic mobility should not be deposition rate-dependent.

In summary, deposition rate most likely affect adatom diffusivity indirectly through another mechanism.

1.7. Alternative mechanism: impurity adsorption

The analysis so far suggests that a changing deposition rate is likely to change the diffusive behavior of adatoms indirectly through a mechanism that is not simply based on the time available for each layer to grow. A possible mechanism is the effect of residual impurity species at a background vacuum of 10^{-7}-10^{-5} mbar that is typical of our experimental setup.

The effect of residual gas species on the diffusive properties and growth properties of a variety of metals have been well-documented. The adsorption of gas molecules on a growing crystal can, facilitate [8,9], modify [6,7], and hinder [5] the diffusion of surface adatoms. In some extreme cases, pressure as low as 10^{-11} mbar have been shown to dramatically influence growth morphology [7]. Given the potentially extreme sensitivity of metal epitaxy to the presence of common gases like O_2, CO, and CO_2, it is not impossible that the current FeCo system is also subjected to their influence. If one of the residual gases in the 10^{-7} to 10^{-5} mbar pressure of our setup played a role in the observed rate dependence (Supplementary Figure 1), it could be because the higher evaporation rate both diluted out the relative concentration of these impurities (Supplementary Figure 2) and reduced their steady-state fractional surface coverage by shortening the time available for them to strike and adsorb onto the surface before the current crystal surface layer is completed and covered.

Testing these hypothesis would require the construction of a UHV deposition system and experimentation with controlled atmosphere across several orders of magnitude in pressure. If residual gas effect were indeed significant, UHV GLAD system may become useful tools for future efforts aiming to generalizing crystal-orientation controlled GLAD nanowire synthesis to other materials.

Supplementary Note 2

2. Engineering for Ultrahigh-Index Nanowires

We point out in Supplementary Methods Section 2.3. that the finite rotation speed of the particular step motor used in our demonstration experiments poses a technical limitation that currently makes it difficult to achieve high-index orientations, such as $\langle 100\,100\,1\rangle$ nanowires. This technical limitation, however, is solvable through engineering of the deposition system.

Here, we will call this the $\langle H\,H\,L\rangle$ nanowire (H for high-index and L for low-index). The task is to increase the ratio $\frac{H}{L}$ by maximizing the relative duration spent growing the H orientation while minimizing that for the L orientation.

Time (relative to the required deposition rate) spent growing H per dwell is constrained by the diameter of the material; the thickness deposited per dwell should be smaller than the nanowire diameter in order to provide material with smooth sidewalls. Otherwise, zig-zag and spiral structures would result. For nanowires with 200 nm diameter, this corresponds to a maximum of about 20 nm deposition in the H direction per dwell. For example, at 3 nm/sec, this is about 7 seconds maximum per dwell along the high-index orientation ($t_H \leq 7\text{sec}$).

Depending on implementation, L is constrained by different factors. For example, in the current implementation based on angle-switched time sharing, time spend growing the lowest index direction during each dwell (t_L) should be substantially larger than the time spent switching between dwell positions (t_{switch}) to minimize the departure of material accretion directions from the rotational symmetry directions of the biaxial film. Based on the results of this study (Supplementary Table 1 and Supplementary Figure 17), we know that $\frac{t_L}{t_{\text{switch}}} \geq 5$ is required. Taken together, the motor used in this study in compatible with a maximum $\frac{H}{L} = \frac{t_H(\text{max})}{t_L(\text{min})} = \frac{7\text{sec}}{5 \times 0.107\text{sec}} = 14.$

In the following subsections, we point interested colleagues to four independent engineering approaches that would enable access to nanowires oriented along ultrahigh-index directions.

2.1. Faster step motor

Faster motors allow for increasing the ratio $\frac{H}{L}$ in the $\langle H\,H\,L\rangle$ nanowire by shortening t_L. Step motors with higher speeds up to 3000 rpm are commercially available. This would correspond to a switching time between dwell positions of 7 msec, compared to 107 msec in this work, to enable access to $\frac{H}{L} = \frac{7\text{sec}}{5 \times 0.007\text{sec}} = 200.$

2.2. Sample rotation-beam shutter synchronization

Another improvement of the angle-switched time sharing implementation consists of blocking the material beam during angle switching. This can be achieved using a rotational chopper (mounted on a second step motor and placed between the sample and the source), similar to those used in optics experiments to create light pulses. Proper synchronization of the chopper step motor with the ϕ angle step motor would allow completely eliminating material deposition from undesirable non-symmetry directions.

This approach would completely eliminate t_{switch} from the equation to make $\frac{H}{L}$ now limited only by how short of a material pulse can be created for growing along the L direction. Reducing t_L through pulsing can be achieved by depositing through a slit, say 1° wide, on the circular chopper. At a rotational speed of 3000 rpm, each pulse would only last $\frac{1°}{50\text{Hz} \times 360°} = 56\text{usec}$. Maximum achievable index ratio thus becomes $= \frac{7\text{sec}}{0.000056\text{sec}} = 125,000$. Producing $\langle 125,000\,1\,1 \rangle$ nanowire would actually have little practical value, since it would be experimentally indistinguishable from a $\langle 1\,0\,0 \rangle$ nanowire (0.0005° offset).

2.3. Ultrahigh vacuum system

Control experiments and discussions in Section 1. suggest that a most plausible explanation for the need for high evaporation rate to achieve single-crystalline growth in the FeCo system is to overwhelm and dilute the effect of impurity molecules by a high flux of the desired atomic species, Fe and Co.

By operating in a UHV evaporation system, it might be possible to achieve single-crystallinity using greatly reduced overall evaporation rates. For example, if impurity adsorption were slowed down by decreasing the background pressure from 10^{-6} torr to, say, 10^{-9} torr, evaporation rate may be reduced to 0.03 /sec while maintaining single-crystalline growth. This would effectively expand the time scale by the same factor of 1000, allowing for $\frac{H}{L} > 10,000$.

2.4. Multiple evaporation sources

An alternative implementation to completely eliminate the effect of t_{switch} is to have multiple physical sources that simultaneously evaporate materials from the symmetry positions (3 in this case). This method would require custom design and construction of dedicated evaporators. The maximum $\frac{H}{L}$ that can be achieved here would be limited by the dynamic range of evaporation rate measurement, necessary for implementing feedback control of the rates. Since the rate along each of the evaporation axes can be measured by independent sensors operating based on different physical principles, very high dynamic range can be achieved. For example, it is possible to measure the arrival of individual

molecules and atoms by nanomechanical resonators [25–27]. Therefore, arbitrarily high-index crystal orientations are achievable with engineering.

Supplementary Note 3

3. A Road Map for Generalization to Other Materials

The growth of arbitrary-$\langle h\,k\,l\rangle$ FeCo nanowires hinges on the rotational symmetry of the underlying biaxial texture that enables symmetry preservation despite anisotropic growth. Consequently, such crystal orientation-controlled nanowire growth is expected to be possible for any material that can be first coaxed to grow as a forest of tilted nanowires embedded within the proper texture. Existing data, though sparse, demonstrate that there is considerable scope for generalization.

3.1. Steps 1: single-crystallinity and biaxiality

A first step in adapting the methodology to a new material consists of inducing single-crystalinity. Modern GLAD systems and processes possess numerous knobs for achieve both of these objectives for a new target material, as detailed in the following.

Many avenues are available for inducing single-crystalline growth, including substrate heating, deposition rate scanning, doping, residual atmosphere control, substrate epitaxial control, and ϕ step-modulation. Raising the substrate to 330°C promotes single-crystalline growth for germanium [28]. The current study further shows that using high deposition rates can also lead to the growth of single-crystals, possibly due to a dilution of nefarious residual gases (Supplementary Note 1, Section 1.7.). In the same line of thought, intentionally adding certain gases to serve as surfactants during growth may also be useful [8,9]. A fourth method consists of adding a small amount of dopant atoms; a literature example are single-crystalline magnesium pillars with drastically improved uniformity and morphology with the incorporation of few percent of titanium dopant [29]. In analogy to more traditional CVD-type growth, substrate epitaxial control can also augment the capability of GLAD, as demonstrated in the growth of crystalline copper on hydrogen-terminated silicon [30].

As a final example, we have applied rapid ϕ stepping to suppress branched growth in materials that have a natural tendency to do so. At $\alpha = 85$ and a high deposition rate of 3 nm/sec, cobalt grows as branched, single-crystalline filaments with a branching angle of 30°. We found that by rapidly switching ϕ to bistable positions separated by 30° significantly suppressed branching to produce parallely-aligned fibers (Supplementary Figure 7). A TKD analysis showed that the fibers are hexagonal single-crystals with a preference for $\langle 1\,0\,0\,0\rangle$ direction along the fiber axis (Supplementary Figure 8b).

These examples together show that multiple handles exist for identifying regions in the parameter space that are compatible with the growth of straight single-crystals.

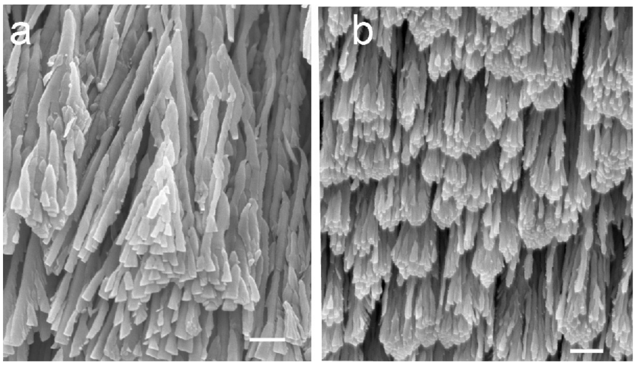

Supplementary Figure 7: Crystallinity control of cobalt nanowire structures. Cobalt structures prepared by stationary GLAD at $\alpha = 85°$ (**a**) and by toggling the substrate between two ϕ orientations separated by 30°. Evaporation rate was 3.0 nm/sec. Scale bars are 200 nm.

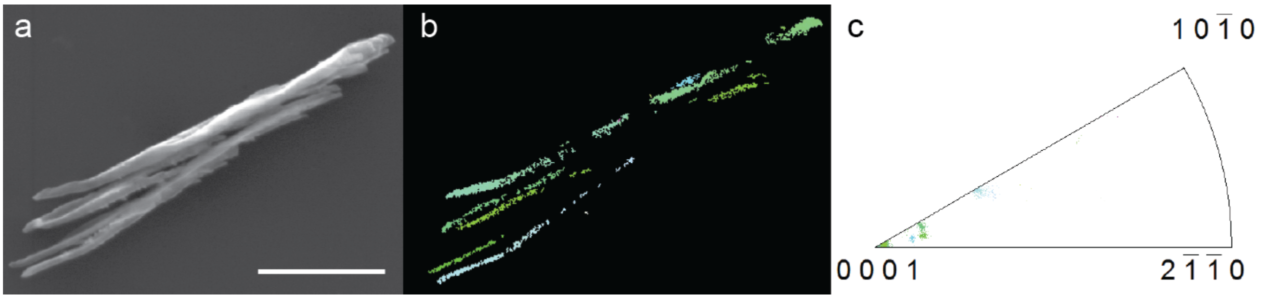

Supplementary Figure 8: TKD analysis of cobalt nanowires. (**a**) SEM micrograph of a bundle of nanowires on carbon TEM grid. (**b-c**) The correpsonding TKD data. Scale bar in (**a**) is 1 μm.

3.2. Step 2: rotational symmetry in the biaxial film

With the basic conditions for single-crystallinity in hand, one can subsequently address rotational symmetry of the biaxial film. Even though biaxiality often arises alongside single-crystallinity due to translational invariance, rotational symmetry does not, in general, automatically ensue. For example, GLAD of molybdenum at a fixed ϕ angle has led to a biaxial texture that lacks any rotational symmetry [31]. The authors showed that ϕ modulation can improve rotation symmetry while preserving the crystal structure. While this example has resulted in vertically aligned wires ($\beta = 0$) that are not useful as a basis for $\langle h\,k\,l \rangle$ growth by vectorial addition, it nevertheless exemplifies the power of ϕ angle modulation as a mean to rotate the symmetry of the biaxial film. Symmetries useful for $\langle h\,k\,l \rangle$ growth can, for example, be induced by a ϕ dwell set that does not have radial symmetry within itself. In addition to ϕ, deviations of the biaxial film from rotational symmetry can be addressed by changing the α angle, which is directly proportional to β for the vast majority of materials investigated to date [32]. The above discussion thus promise considerable opportunity for generalizing arbitrary $\langle h\,k\,l \rangle$ growth demonstrated in the current work on a FeCo model system to other material systems.

Supplementary Note 4

4. Avenues for Future Research in Single-Crystalline GLAD

The current work introduces a method to control the crystallinity of nanowires produced by GLAD. Equally important for any method for nanomaterial synthesis are controls over nanowire density, on-chip placement, diameter, and aspect ratio. In this section, we discuss future research directions for developing these added control capabilities.

4.1. Density and on-chip placement

The density and placement of GLAD pillars can be completely controlled through the use of a two-step electron beam lithography seeding method [33]. Despite the rather preliminary nature of this report, in which imperfect technical execution (such as over-exposure in the process step in Fig. 1d-e, evident in data in Fig 2b) of the proposed concept led to broadening of the pillars in the design locations, optimization of fabrication procedure would enable arbitrary and simultaneous control over density, placement, and crystal orientation.

4.2. Diameter control

Diameter control is a multi-faceted task involving intra-nanowire uniformity, inter-nanowire uniformity, and the average diameter.

4.2.1. Intra-nanowire uniformity

Within the realm of amorphous and polycrystalline pillars, intra-wire uniformity can be controllable by ϕ-angle modulation techniques ([34, 35], Chapter 3 in [36]) techniques to decrease the effect of stochastic fluctuation in self-shadowing areas. Since the control of crystal orientation also relies on ϕ-angle modulation, the extent to which this approach can be combined with single-crystal GLAD remains to be explored. It is, however, likely that substrate swing with small ϕ-angle excursions ([36]), Figure 3.16 c) can be compatible with crystal orientation control that uses much larger ϕ excursions.

Within the realm of single-crystal pillars, a recognized mechanism for intra-wire diameter variation is gradual increase of wire diameter with length due to adatom diffusion across the Ehrlich-Schwoebel (ES) barrier [37]. Therefore, adjusting the available thermal energy with respect to the ES barrier heights (diffusion down single or multiple steps have different activation energies) can be used as a handle to tune the steady-state diameter during the growth of single-crystalline nanowires ([37],

Fig. 3). It is conceivable that dynamically tuning the substrate temperature during a deposition run could be used to modulate intra-wire diameter ([38], Fig. 3 and Fig. 4).

4.2.2. Inter-nanowire uniformity and average diameter

Inter-nanowire uniformity and mean pillar diameter can be controlled by lithographic seeding ([39], Fig. 8) or by the effective diffusion length of the adatoms, adjustable by substrate temperature and by evaporation rate [38, 40].

4.3. Aspect ratio

Maximum-achievable aspect ratio is limited only by engineering details of the evaporation chamber. Two factors contribute to this parameter, corresponding to the maximum length and the minimum diameter.

4.3.1. Maximum length

The duration of the evaporation run establishes the maximum nanowire length. In our setup, the maximum is limited by the fact that there is a single quartz crystal rate monitor inside the chamber. This quartz crystal balance fails after a period of material deposition, and opening the chamber to exchange to a new rate monitor effectively terminates the deposition run. Ebeam evaporator chambers with multiple quartz crystal microbalance exist and would allow longer nanowires to be grown. Eventually, the amount of evaporation material in the source may aloso become limiting. Modern commercial evaporators can host >8 hearths and would enable wires with length above 1 cm to be grown, provided, of course, that a sufficient number of quartz crystals monitors could be operated in tandem and/or exchanged *in situ* via specially designed load-lock.

4.3.2. Minimum diameter

Diameter control has been discussed previously above.

While the current study involved (unintentionally) producing nanowires with maximum aspect ratio of about 30, increasing the length and decreasing the diameter, as discussed above, would enable substantially increasing this parameter by at least an order of magnitude.

Supplementary Note 5

5. Ultimate Limits in Crystal Orientation Control

Supplementary Note 2. discusses engineering approaches to enable high-precision control of nanowire crystal orientation. In this section, we discuss the ultimate limit that can be achieved through engineering. We conduct this discussion by analyzing two physical details of the evaporation process. The first process we consider is the finite duration in time for the evaporated atoms to reach the substrate. The second process we consider is the fact that the sticking coefficient of arriving atoms on the tips of the growing nanowires is not unity. We first analyze each of the two processes based on an assumption of a continuous stream of matter. We show that if matter were infinitely divisible to provide continuous vapor streams, statistical variations do not arise, neither from the finite vapor travel time nor from a none-unity sticking coefficient. Instead, the discreteness of atoms leads to fundamental directional control variabilities on the order of 0.01%-0.1% through a statistical fluctuation in the rate of deposition.

5.1. Finite time delay between the evaporation and the deposition of material

Consider a sharp square pulse of vapor, of duration δt and carrying an amount of material $M(\delta t)$, that is released at time $t = 0$ from an ebeam source. This packet of matter travels toward the substrate placed at distance L away. The material in $M(\delta t)$ will display some distribution in velocities described by some probability function, $g(v)$. Regardless of the exact shape of $g(v)$, a detector placed at the site of the substrate would observe the arrival of matter between times t_f and t_s $(0 < t_f < t_s)$, where $t_f = \dfrac{L}{v_f}$ and $t_s = \dfrac{L}{v_s}$, with v_f and v_s being, respectively, the highest and the lowest velocities for which $g(v) \neq 0$. The sharp square pulse of vapor we consider has a duration that is small compared to the dispersion in arrival time: $\delta t \ll t_s - t_f$.

There are a few things to notice about the relevant velocities and times. In ebeam evaporation, atomic speeds are generally on the order of 10^3 m/sec [41], and are distributed according to some distribution, such as the Maxwell-Boltzmann distribution. This means that the relevant transit times in a typical evaporator are below 1 msec. We also have to note that the velocities of atoms do change during the trip from the source to the substrate as a result of inter-particle interactions, so that we can take v introduced in the previous paragraph as the average velocities for the vapor when traveling between the source and the substrate.

The material detector placed at the substrate would register some function, $m(t) \neq 0$, when $t_f < t < t_s$, and $m(t) = 0$ outside of this time window. Notice that the probability densities $\dfrac{m(t)}{M(\delta t)}$ and

$g(v)$ are uniquely mapped to each other through a transformation of the velocity and arrival time random variables. And the integral of $m(t)$ between t_f and t_s gives $M(\delta t)$.

5.1.1. Continuum limit

When matter were assumed to be continuous, it would be possible to reduce the pulse width towards zero ($\delta t \to 0$) without affecting the shapes of $m(t)$ and $g(v)$. At any instant in time, t_{now}, material arriving at the substrate would be an integral (linear superposition) of partial contributions from an infinite number of adjacent pulses previously released from the source at between times $t_{\text{now}} - t_s$ and $t_{\text{now}} - t_f$. Notice that the fractional contribution from a particular pulse released at time $t_{\text{now}} - t$ is described by $m(t_{\text{now}} - t)$. The rate of material arrival at the substrate would be the integral of such partial contributions between $t_{\text{now}} - t_s$ and $t_{\text{now}} - t_f$. This integral gives $M(\delta t)$. Thus, the rate of material arriving at the substrate would exactly equal the rate of evaporation from the source in the continuum limit, even in the presence of an arbitrarily shaped vapor velocity distribution, $g(v)$. In this case, control precision is limited by engineering.

5.1.2. The reality of discrete atoms

The situation is different in reality because matter is atomically discrete. As a result, the step of letting $\delta t \to 0$ while hoping to conserve the shapes of $m(t)$ and $g(v)$ no longer hold; when δt were made sufficiently small, at a certain point, one would observe either the landing of at most a single atom during δt or none at all. Averaged over time, this is equivalent to a probability of arrival: $p(\delta t)$, a quantity proportional to δt through multiplication by the evaporation rate R (atoms/second). Therefore, during time t_{dwell} when one of the 3 symmetry positions (as in the main manuscript) is facing the source, there will be a variance, as per binomial distribution, in the total number of arriving atoms, N: $\delta N^2 = np(\delta t)[1 - p(\delta t)]$, where $n = \frac{t_{\text{dwell}}}{\delta t}$ is the number of time periods. Substituting in n and taking the limit $\delta t \to 0$ show that the standard deviation is proportional to $\sqrt{R \times t_{\text{dwell}}} = \sqrt{N}$.

For a typical 1-second dwell at the 3 nm/sec used in our experiment, there is about 10^5 nm^3 of FeCo material, or 5E6 atoms, deposited at the end of each nanowire. The standard deviation in the number of atoms arriving will be on the order of 2E3. Or 0.05% of the total. However, when dwell time gets shortened to around 10 msec with the implementation of a faster step motor, as in the fabrication of ultra-high-index nanowires (Supplementary Note 2.), the statistical fluctuation would grow to 0.5%. This is a fundamental limit imposed by the discreteness of matter and the nanometer dimension of the target material. Since multiple rotation cycles are used to grow a complete nanowire, averaging over the length would reduce the statistical directional uncertainties to the 0.01-0.1% level at dwell

times on the order of 10 msec.

5.2. None-unity sticking coefficient

The analysis is similar to the above. Under the assumption of continuous material flux, a sticking coefficient $p_s \neq 0$ decreases the effective evaporation rate to $R_e = p_s R$, without introducing a new mechanism to increase directional uncertainty. In contrast, the reality of atomic discreteness introduces statistical variation in the number of sticking atoms. The magnitude of this uncertainty is smaller by at least a factor of 2 compared to the variation introduced by the previously discussed mechanism, as per the binomial distribution and by the fact that sticking coefficients under ebeam conditions are very close to one.

In summary, the fundamental limit of precision in GLAD crystal orientation control for single-crystal nanowire fabrication is on the order of 0.01% to 0.1%. This limit is a result of the atomic discreteness of matter and the nanoscale dimensions of the target objects.

Supplementary Methods

1. Experimental System

1.1. Motor control hardware and software

We constructed a standard GLAD setup by implementing a computer-controlled ϕ-rotation sample stage with manually adjustable α inside a commercial electron beam evaporator (Pfeiffer Classic 500) (Supplementary Figure 9). Sample ϕ rotation was achieved using a step motor (Oriental Motor, model: LCMK245AP). The motor stepping motion (0.1125° step size) was activated by sending a square waveform (10 kHz, 50% duty cycle) generated by an arbitrary waveform generator (HP Agilent Keysight, 33120A) to the motor driver (24 VDC input micro step driver). The stepping direction was chosen via voltage control signals sent to the motor driver from a National Instruments PCI DAQ card. Timing and amplitudes of motor movements were centrally controlled using custom software written in Labview 2009.

1.2. Motor thermal anchoring

The sample stage is not temperature-regulated. This fact behooves us to minimize thermal disturbance to the sample caused by extended operation of the motor. This is especially important because heat dissipation by convection is absent in a vacuum environment. To achieve efficient thermal anchoring, we constructed the motor mount and the α rotation arm from high-purity copper plates (1 cm thickness) and cylinder (4 cm-diameter), respectively. The α rotation arm was furthermore heat-

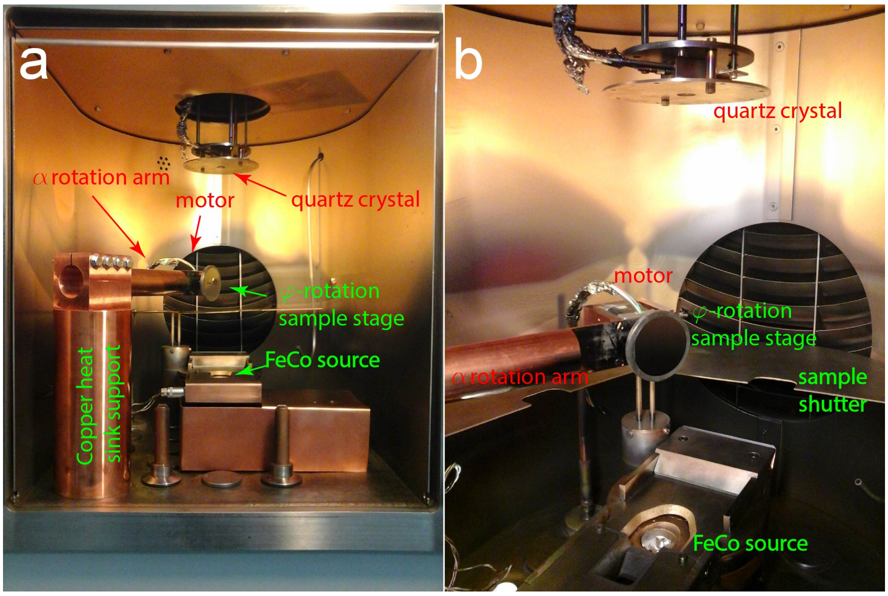

Supplementary Figure 9: GLAD setup. (a) Overview of the evaporator chamber with the GLAD sample stage and copper thermal anchoring post. (b) A zoom-in view showing details of the source, sample, and quartz crystal.

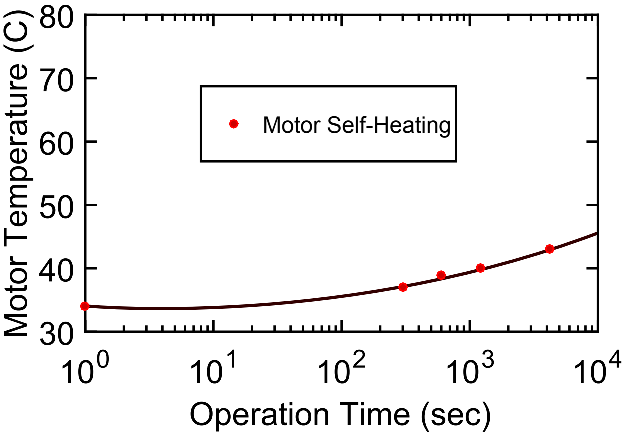

Supplementary Figure 10: Heating caused by step motor operation. The temperature of the motor was monitored as it was continuously operated in vacuum. The electron beam was off and material deposition was not applied in this control experiment. Solid line is a guide to the eye.

sunk against a 10 cm-diameter, 30 cm-tall cylindrical copper block in contact with the water-cooled floor of the chamber (Supplementary Figure 9a).

The efficiency of the thermal anchoring for the motor was experimentally verified by its continuous operation in vacuum for over 1 hour. The resulting temperature of the motor was monitored using thermocouple and is plotted in Supplementary Figure 10. In light of these results, all ϕ-variable depositions were conducted after warming up the motor for 30 minutes. Given that a typical deposition run lasts ≈1200 seconds, motor operation heats the sample by no more than a couple of degrees Celsius.

1.3. Evaporation material

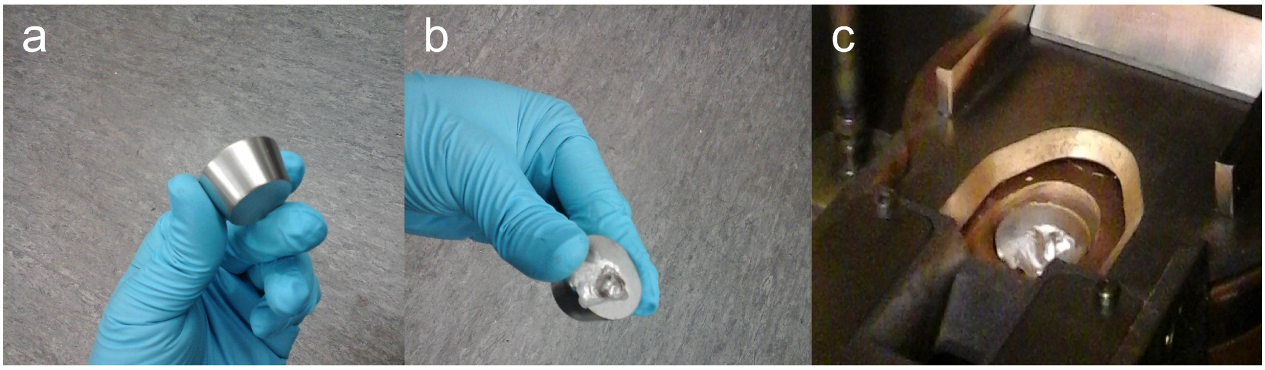

Supplementary Figure 11: Monolithic VIM starter source for high-rate evaporation. (a)-(b) backside and front-side views of a (used) bulk FeCo VIM starter source. (c) The source is loaded into the evaporator copper pocket without liner.

To achieve steady and high deposition rates over tens of minutes, it is crucial to use bulk FeCo (and Co: Supplementary Note 3.) materials that had been alloyed by vacuum induction melting (VIM). Otherwise, dissolved gases and uneven heating could trigger large rate fluctuations, and even sudden

explosion of the source material during evaporation. Iron/cobalt starter sources (VIM Fe/Co 65/35 Atomic %, 99.95% pure) machined into the shape of standard evaporation crucibles (29.3 mm top OD $\times$ 22 mm bottom OD $\times$ 15 mm height) were purchased from Kurt J. Lesker Company (Supplementary Figure 11a-b). The starter source was directly fitted into the cooled copper pocket of the ebeam system without using a liner (Supplementary Figure 11c). Prior to loading the FeCo source, the copper pocket was meticulously cleaned by polishing with fine sand paper and isopropanol-drenched Kimwipe, until the interior shone like newly machined, reddish copper. For reproducible results, we found it important to avoid contamination of the source material by also thoroughly cleaning the sample shutter and other parts of the system where debris of other evaporated materials could peel off and accidently fall into the source. Starter source was wiped clean with isopropanol-drenched Kimwipe before loading into the ebeam pocket.

Gloves were worn during the entire chamber preparation procedure and changed promptly when contaminated by visible marks.

2. Experimental Procedures

2.1. Substrates

Substrates used in this study include wafers of Si(100)/Si(111) (native oxide and Si-H terminated), sapphire, glass, kapton tape, and coatings of PMMA and ZEP on flat Si(100). Native oxide-bearing silicon, sapphire, glass, and Kapton substrates were cleaned in IPA and blown dry with nitrogen. PMMA and ZEP coatings were used after spin coating. Hydrogen-terminated silicon substrates were mounted into the system and pumped down to vacuum within 30 seconds of etching in 1% HF.

As during the preparation of the source material, gloves were worn at all times during the handling and loading of sample substrates.

2.2. Chamber vacuum preparation

Chamber pressure before the start of deposition runs was in the low 10^{-6} mbar range. Before each nanowire growth evaporation, the FeCo source was first cleaned by evaporating away some material with the substrate shutter still closed. This step usually led to a slight increase in chamber pressure. Then, the chamber was further pumped by evaporating Ti at a rate of 1 nm/sec over 2 minutes (still with shutter closed). The chamber pressure dropped down into the low 10^{-7} mbar range. Immediately after the Ti pumping step, the nanowire growth step was conducted by opening the shutter after FeCo evaporation rate had stabilized to the target value. The electron beam was held fixed with neither wobbling nor scanning motions to best approximate a point source. Chamber pressure during nanowire growth increased slowly from the 10^{-6} mbar range into the low 10^{-5} mbar range.

2.3. Orientation-controlled nanowire growth

For most of the work presented in the main text, an evaporation rate of 3.0 nm/sec at the sample was used. Because of a tooling factor of 5 between the sample and the quartz crystal, the measured, control set-point rate was 0.60 nm/sec.

FeCo nanowires along crystal orientations $\langle h\,k\,l\rangle$ were grown by periodically orienting the 3 azimuthal directions separated by 120° toward the source, such that the relative dwell times along each of the directions were in the ratios of $h : k : l$ (Fig. 3, main text). Samples presented in this study were prepared using dwell times summarized in Supplementary Table 1.

The step motor could only be reliably actuated at stepping frequencies less than about 15 kHz when starting from still. We therefore adopted a stepping frequency of 10 kH. This technical limitation in

rotational toggling speed caused 107 msec to be spent in intermediate orientations between toggling events. The percentage of time spent in the 3 desired, rotationally symmetric directions is listed in the last column, and should ideally approach 100%.

Nanowire Orientation $\langle h\,k\,l \rangle$	Position 1 (msec)	Position 2 (msec)	Position 3 (msec)	Toggle Time (msec)	Time at the 3 Stationary Positions (%)
$\langle 1\,1\,0 \rangle$	500	500	0	107	79
$\langle 1\,1\,1 \rangle$	1000	1000	1000	107	89
$\langle 2\,1\,0 \rangle$	1000	500	0	107	86
$\langle 3\,1\,0 \rangle$	1500	500	0	107	89
$\langle 3\,2\,0 \rangle$	1500	1000	0	107	91
$\langle 3\,2\,1 \rangle$	1500	1000	500	107	89

Supplementary Table 1: Dwell times at each of the 3 equivalent azimuthal orientations and relevant parameters for the presented nanowires.

2.4. Spiral growth parameters

The spiral sample presented in Fig. 4d-4f of the main text was fabricated using the following recipe steps, executed sequentially in one continuous deposition run (Supplementary Table 2). The design starts with a $\langle 1\,1\,1 \rangle$ post normal to the substrate, followed by 6 branches along $\langle 1\,0\,0 \rangle$ and $\langle 1\,1\,0 \rangle$ directions that make a hexagon when projected onto the substrate plane. Another $\langle 1\,1\,1 \rangle$ segment follows before 4 segments with different turning angles cap the structure. The standard deposition rate of 3.0 nm/second was used and the dwell times used for each of the crystal orientations are as listed in Supplementary Table 1.

Segment Number	Segment Orientation $\langle h\,k\,l \rangle$	Segment Length (nm)	Segment Time (sec)
1	[1 1 1]	600	200
2	[1 0 0]	300	100
3	[1 1 0]	300	100
4	[0 1 0]	300	100
5	[0 1 1]	300	100
6	[0 0 1]	300	100
7	[1 0 1]	300	100
8	[1 1 1]	600	200
9	[1 0 0]	300	100
10	[1 2 0]	519	173
11	[0 1 1]	300	100
12	[1 0 2]	519	173

Supplementary Table 2: Orientation and deposition thicknesses for the segments of the spiral in Fig. 4

2.5. Hybrid nanowire fabrication

FeCo-Al_2O_3 Atomic layer deposition (ALD) of alumina around FeCo nanowires were performed inside a commercial ALD system (Picosun Sunale R-150B). Within 1 hour of the fabication of FeCo nanowires by the standard procedure described above, the sample was loaded into the ALD chamber pre-equilibrated to a temperature of 100°C. Al_2O_3 was deposited at a relatively low process tem-

perature of 110°C as a cautionary measure to prevent any potential oxidation of the FeCo material. Post-coating SEM inspection and magnetization measurements confirmed the absence of any magnetic material degradation during coating at this temperature. $AlCl_3$ and water pulses were 100 msec and the chamber pumping step was 4 seconds. 50 cycles were applied for a nominal coating thickness of 5 nm.

FeCo-Nb, FeCo-Ho, and FeCo/MgO/FeCo were all fabricated based on a FeCo $\langle 1\,0\,0 \rangle$ base. All hybrids were each made in the same evaporation run without breaking vacuum. As a result, all required materials for the heterostructure, as well as titanium for chamber cleaning purposes, were loaded into one of the available pockets of the evaporator prior to pumping down to vacuum.

<u>Nb-FeCo</u> After the deposition of the FeCo segment at a deposition rate of 3 nm/sec, the shutter was closed and the evaporation source was switched to niobium. Niobium was deposited at a rate of 0.5 nm/sec until the desired thickness was reached. For the particular sample presented in Fig. 5 of the main text, the nominal Nb thickness was 20 nm. The slower rate for Nb deposition was chosen due to the much higher ebeam power that is needed to evaporate this material.

<u>Ho-FeCo</u> Due to the relative high reactivity of holmium, the vacuum chamber was cleaned, with the sample shutter closed, by a round of Ti evaporation (Section 2.2.) after the FeCo segment. Holmium was deposited at at rate of 3 nm/sec to the desired thickness. The sample presented in Fig. 5 of the main text had a nominal Ho thickness of 300 nm.

<u>Si-FeCo</u> Procedures for producing the samples shown in Supplementary Figure 27 vary in terms of whether the FeCo and Si portions were produced with a static ϕ-angle or with uniform ϕ-rotation at 12 rpm. Different thicknesses for each of the layers were used (See Supplementary Figure 27 for details). Deposition rates were 3 nm/sec for both materials. Silicon layer was deposited immediately after FeCo layer without an intervening Ti pumping step.

<u>FeCo/Ho/FeCo</u> After depositing the FeCo base, the sample stage was subjected to a one-time ϕ-rotation of 120°. MgO was evaporated at a rate of 0.1 nm/sec until a 5 nm layer was deposited. Subsequently, source was changed back to FeCo and a 100 nm layer was evaporated without ϕ movement at 3 nm/sec.

3. Nanowire Manipulation

3.1. Detachment from growth substrate

To use the grown FeCo nanowires in applications such as scanning probe microscopy or solution-phase micromanipulation, one must be able to detach the nanowires from the growth substrate. Supplementary Figure 12 shows examples of $\langle 1\,0\,0 \rangle$ nanowires transferred from the growth substrate to lying flat on a fresh silicon wafer. The transfer was achieved by picking up the nanowires using a piece of Gel-Pak and subsequently stamping onto the receiver surface.

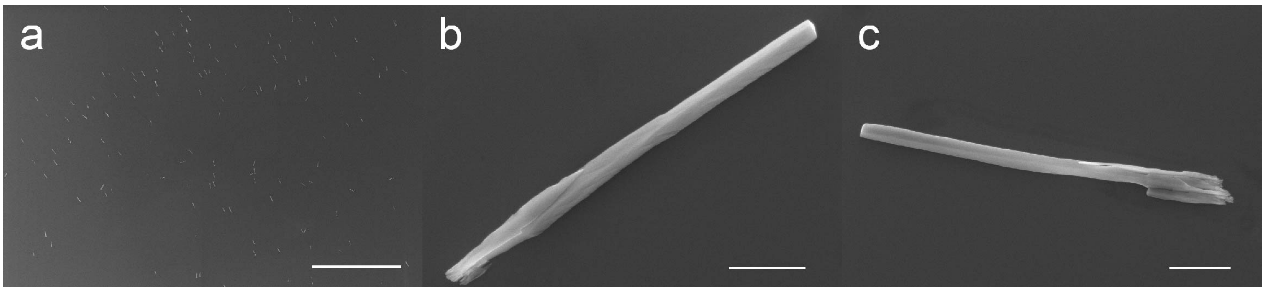

Supplementary Figure 12: Isolation of FeCo nanowires from growth substrate. Overview of a receiver surface (**a**) and zoom-in views on two individual FeCo nanowires (**b**-**c**). Scale bars are 50 μm in (**a**) and 500 nm in (**b**-**c**).

3.2. Nanowire micromanipulation

FeCo nanowires transferred onto a flat substrate (Supplementary Figure 12) can be handled by micromanipulation and re-positioned with sub-micron precision. Supplementary Figure 13 provides an example where a nanowire is loaded onto the tip of a ultrasensitive silicon cantilever for use as a magnetic scanning tip.

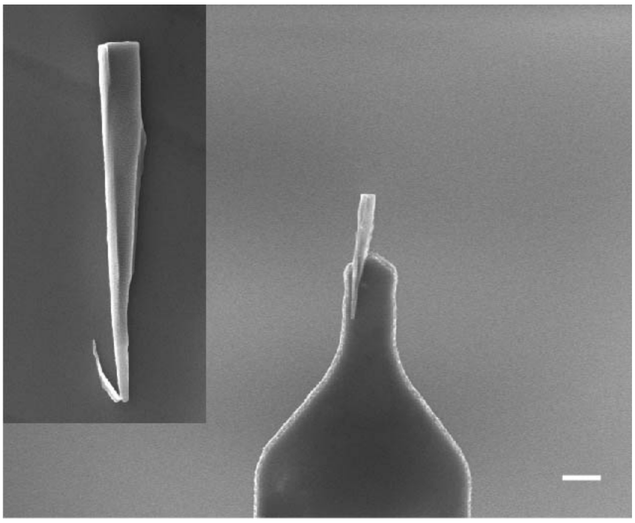

Supplementary Figure 13: Micromanipulation of a FeCo Nanowire. A $\langle 1\,0\,0\rangle$ FeCo is transferred from a flat substrate (inset) to the tip of a silicon cantilever. Scale bar is 1 μm.

4. Characterization of Nanowire Crystallinity and Structure

The crystal structure and crystal orientations of the FeCo nanowires were characterized by high-resolution transmission electron microscopy (HRTEM) and by transmission Kikuchi diffraction (TKD). HRTEM images furnish unequivocal evidence of local single-crystallinity by providing atomic-resolution images of the nanowire crystal. TKD provides complementary, global, and population-level information by enabling mapping over large sample areas containing multiple nanowires.

Material composition and structure of hybrid nanowires were determined by energy-dispersive X-ray spectroscopy (EDX).

4.1. High-resolution transmission electron microscopy

Transmission electron microscopy (TEM) analysis of the nanowires were performed on a FEI Tecnai F30 instrument operated at 300 kV. HRTEM analysis was performed on $\langle 1\,0\,0 \rangle$ wires oriented into the $[1\,0\,0]$ zone axis.

4.2. Transmission Kikuchi diffraction

TKD is a variant of electron backscatter diffraction (EBSD), in which rapid indexing of electron backscatter diffraction patterns enable crystallographic orientation mapping of bulk sample surface areas. This type of analysis is also known as orientation imaging microscopy [42]. In practice, a suitably positioned forescatter electron detector records orientation contrast images with high angular sensitivity, under the same conditions as for standard scanning electron microscopy (SEM). The spatial resolution of EBSD is limited by the scattering of the primary electron beam (10-30 keV) at shallow incident (20°) to the surface of a bulk sample. Lateral resolution of about 20nm×50nm can be achieved by conventional EBSD [43].

TKD capitalizes on standard EBSD hardware and software, but is applied to nanomaterials using a sample-detector geometry that avoids scattering-induced resolution degradation. Samples suitable for TKD analysis include nanoparticles, nanowires, suspended thinfilms, etc, that can be placed onto a TEM grid held perpendicularly to the incident electron beam (Supplementary Figure 14a). By minimizing beam-sample interaction volume, this isotropic geometry achieves sub-10nm spatial resolution [44, 45].

To analyze the nanowires, we used a TKD setup built using commercial components (Supplementary Figure 14a) including a SEM (FEI Quanta200F), a scanning transmission electron microscopy (STEM) sample holder (FEI) enabling a working distance of 5 mm, and an EBSD detector (Ametek-EDAX Hikari) with FSD diode attached. Sample nanowires or spirals were deposited onto TEM grids

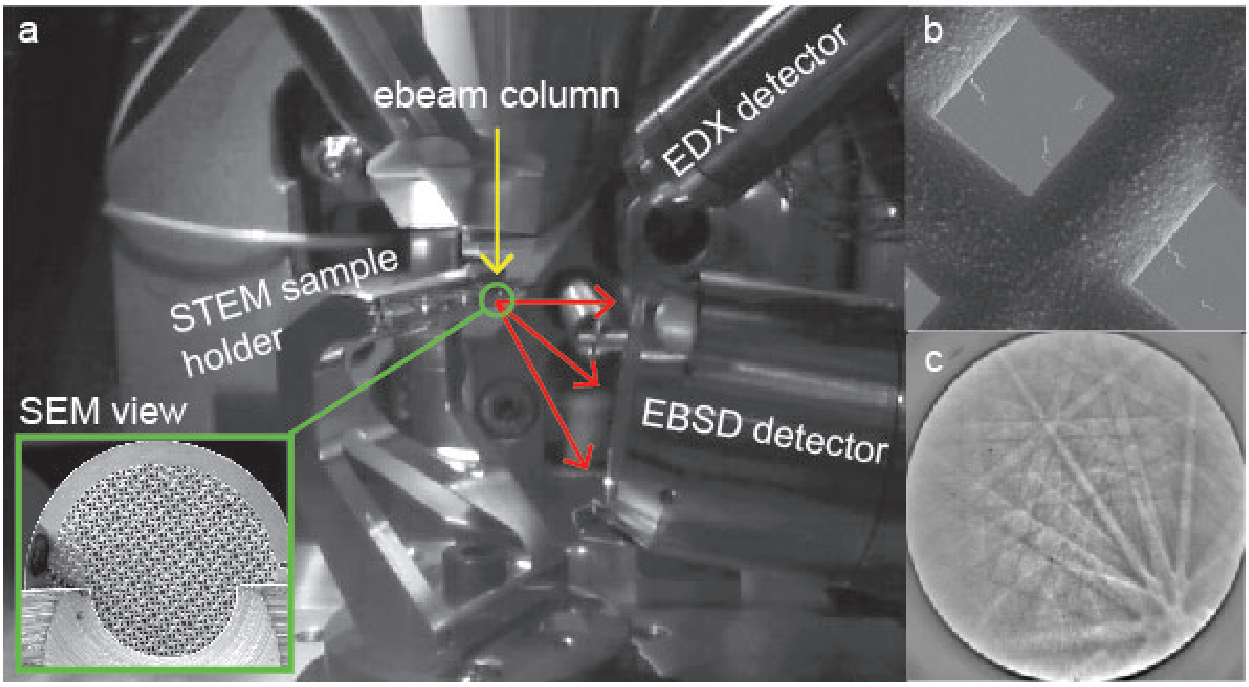

Supplementary Figure 14: Transmission Kikuchi Diffraction setup. (**a**) CCD camera picture of the analysis chamber. Inset shows a scanning electron micrograph of a TEM grid on which sample nanowires or spirals are scattered. (**b**) A zoom-in SEM view of a TEM sample grid with single-crystal FeCo spiral structures. (**c**) Real-time electron diffraction image recorded on the EBSD detector, from a single point on the sample.

by dragging the carbon film-side of the copper grids lightly over the GLAD sample surface. This procedure led to the deposition of individual wires or spirals onto the carbon film (Supplementary Figure 14b). The TEM grids were clamped into a scanning STEM sample holder (Supplementary Figure 14a-Inset). It was important to make sure that the carbon film side was facing down, so that scattered electrons could reach the detector below without being shadowed by the copper grid meshes.

Data were collected with a ebeam voltage of 30 keV, an aperture of 40 μm, a spot size of 5, pixel exposure between 10-22 msec, and corresponding frame rate between 45-100 Hz. Stepsize during area mapping varied between 5nm to 20nm, depending on the size of the scan area. Typical TKD raw data for a single pixel as captured by the detector is shown in Supplementary Figure 14c. Before scanning over areas, such single-pixel diffraction patterns were first taken to allow the software to determine the crystal structure of the crystal. During scanning, the software can then determine in realtime the orientation of the current pixel with respect to the measurement frame by fitting the theoretically expected diffraction maxima (lines) to the data. Examples of typical data are presented in Fig. 4 of the main text. Additional examples for other types of nanowires not discussed in the main text can be found in Supplementary Figure 25 and Supplementary Figure 8.

4.3. Energy-dispersive X-ray spectroscopy of hybrid nanowires

EDX was performed in the same SEM instrument (FEI Quanta200F) as that used for the EBSD analysis. The electron beam was operated at 30 keV and the detector was placed to collect emitted X-ray radiation at 35° takeoff angle (Supplementary Figure 14a). The magnification was ×100,000 and dwell-time was 200 msec for 3 nm × 3 nm pixels (Nb-FeCo, Fig. 5d) and for both 4 nm × 4 nm pixels (Ho-FeCo, Fig. 5e).

4.4. Additional HRTEM data

SEM inspections of the nanowires (Fig. 3) show visible surface roughness. We inspected the edges and a corner of a ⟨1 0 0⟩ nanowire to gain more insights into the surface structure of these nanowires. The images show that there is a 2-3 nm crystalline surface layer with structure different from the bulk FeCo underneath. This could be a surface oxide layer that grew over a period of two years since the time of fabrication and the time of the HRTEM inspection. The results also show that the surface roughness is not a result of polycrystallinity.

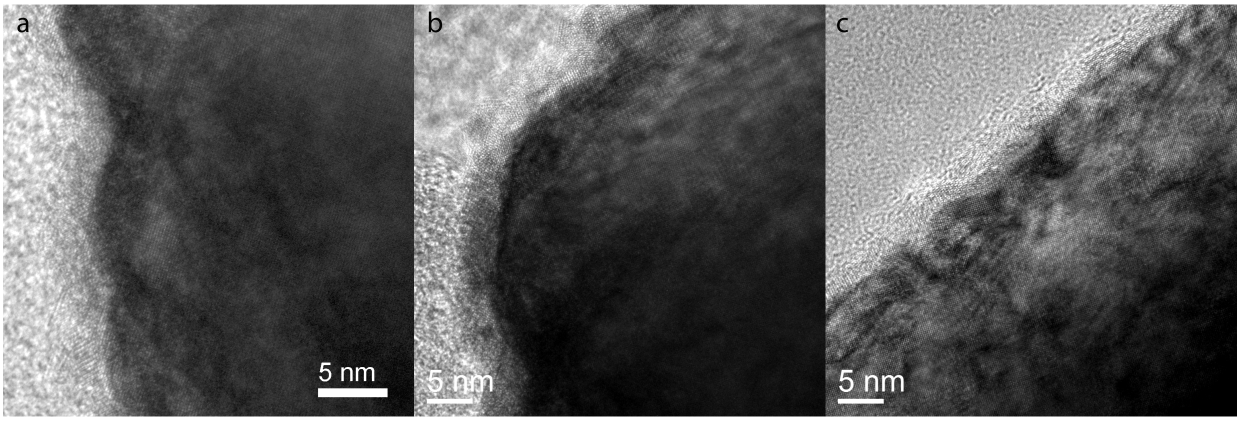

Supplementary Figure 15: HRTEM of nanowire surface. (**a**) The top, growing tip of a ⟨1 0 0⟩ nanowire. (**b**) A top corner of a ⟨1 0 0⟩ nanowire. (**c**) Sidewall of a ⟨1 0 0⟩ nanowire.

4.5. Additional EDX data

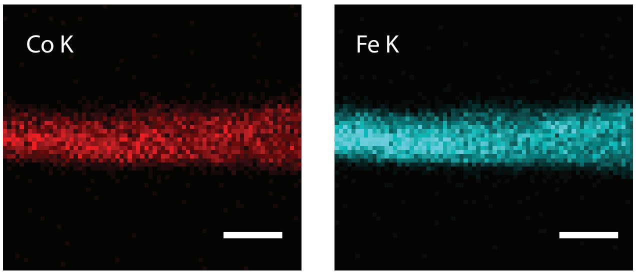

Supplementary Figure 16: Iron and cobalt EDX signals from a nanowire segment. Scale bars are 200 nm.

5. Additional FeCo Growth Data

5.1. β angles

The β angles of nanowires within a biaxial film determines the crystal orientation along the nanowire axis. We have measured the β angles of $\langle 1\,0\,0\rangle$, $\langle 1\,1\,0\rangle$, $\langle 1\,1\,1\rangle$, and $\langle 3\,2\,1\rangle$ nanowires by cross-sectional SEM and the data are summarized in Supplementary Figure 17.

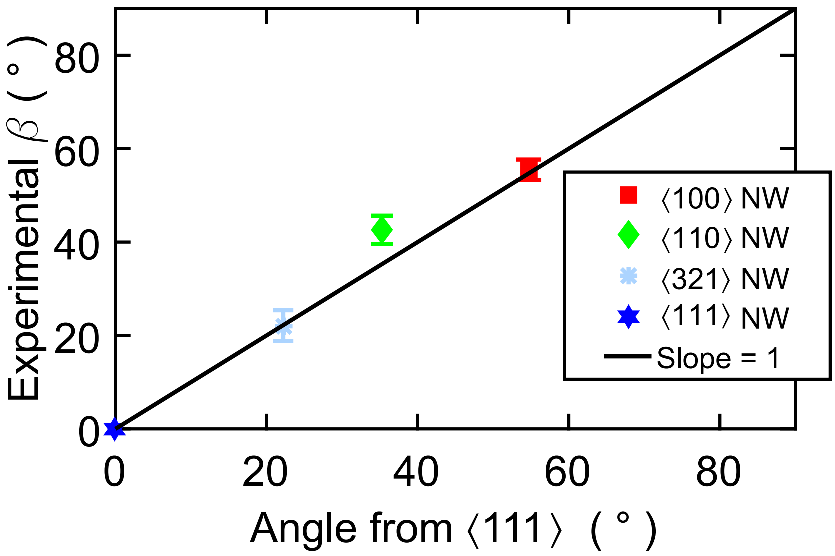

Supplementary Figure 17: β angles of nanowires. Measured β angles are plotted as a function of theoretically-expected angles, within a cubic lattice, between the target orientations and the $\langle 1\,1\,1\rangle$ direction. A slope of unity is consistent with $\langle 1\,1\,1\rangle$ being collinear with the substrate surface normal.

The data are consistent with a $[1\,1\,1]\{1\,1\,0\}$ biaxial texture. We observe a larger-than-expected tilt for the $\langle 1\,1\,0\rangle$ sample, which is likely caused by a significant fraction of time (21%) spent toggling between the ϕ dwell positions for that particular sample (Supplementary Table 1). Evaporation at intermediate ϕ angles is intuitively expected to increase β by the same principle of vector addition. We therefore believe it to be a desirable technical improvement to minimize switch time between ϕ dwell positions as much as possible (Supplementary Note 2.). Such advances would decrease the amount of material deposited from intermediate orientations that can lead to degradation of crystalline order due to disturbance to the rotational symmetry during steady-state growth.

5.2. Large-area SEM views

Large-area views of $\langle 1\,1\,0\rangle$, $\langle 1\,1\,1\rangle$, and $\langle 3\,2\,1\rangle$ samples are provided in Supplementary Figure 18.

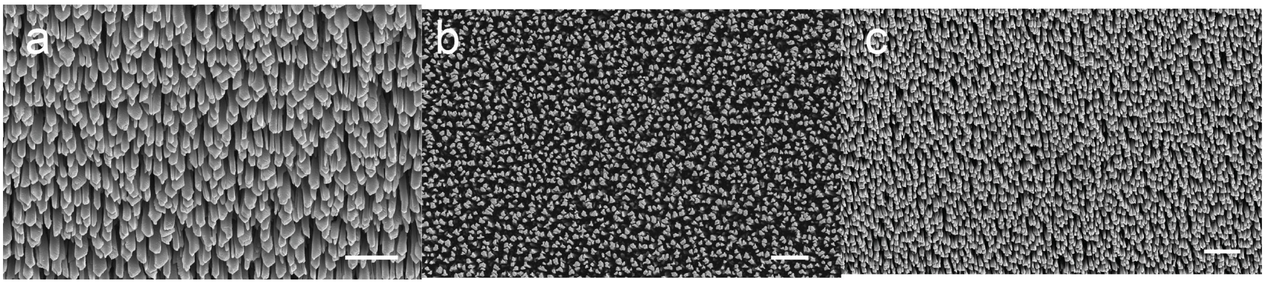

Supplementary Figure 18: Large-area SEM micrographs of (**a**) $\langle 1\,1\,0\rangle$, (**b**) $\langle 1\,1\,1\rangle$, and (**c**) $\langle 3\,2\,1\rangle$ samples viewed perpendicularly to the substrate. Scale bars are 2 μm.

5.3. $\langle 2\,1\,0\rangle$, $\langle 3\,1\,0\rangle$, and $\langle 3\,2\,0\rangle$ nanowires

SEM micrographs of nanowires with several crystal orientations not presented in the main text are provided in Supplementary Figure 19.

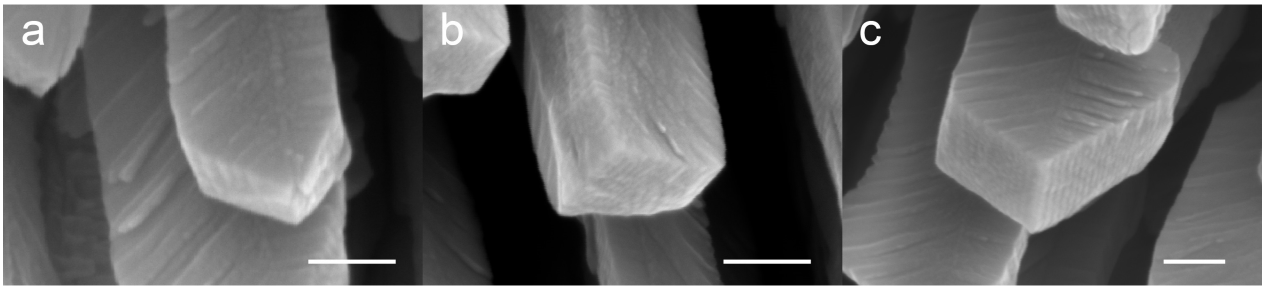

Supplementary Figure 19: SEM micrographs of FeCo nanowires with several additional crystal orientations. (**a**): $\langle 2\,1\,0\rangle$, (**b**):$\langle 3\,1\,0\rangle$, and (**c**): $\langle 3\,2\,0\rangle$. Scale bars are 100 nm.

5.4. Spiral sample

SEM micrographs of the cross sectional views of the spiral sample are provided in the following.

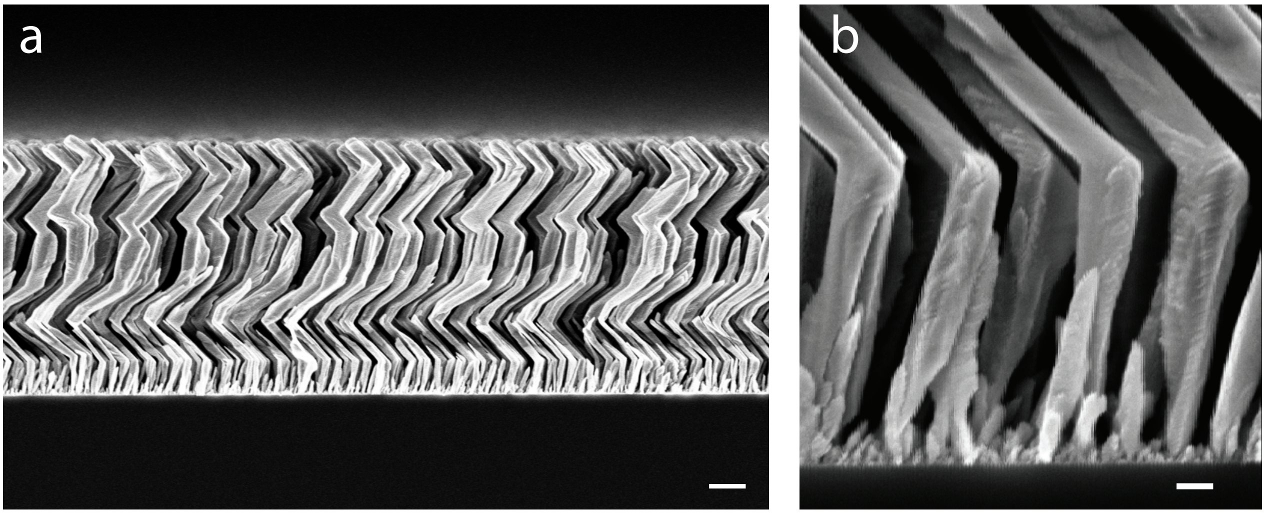

Supplementary Figure 20: SEM micrographs of FeCo spirals, viewed along the cross-section of the substrate. (**a**) Overview. (**b**) Zoom-in view at the the base of the spiral near the substrate. Scale bars are 1 μm and 100 nm.

5.5. FeCo-MgO-FeCo Trilayer Tip

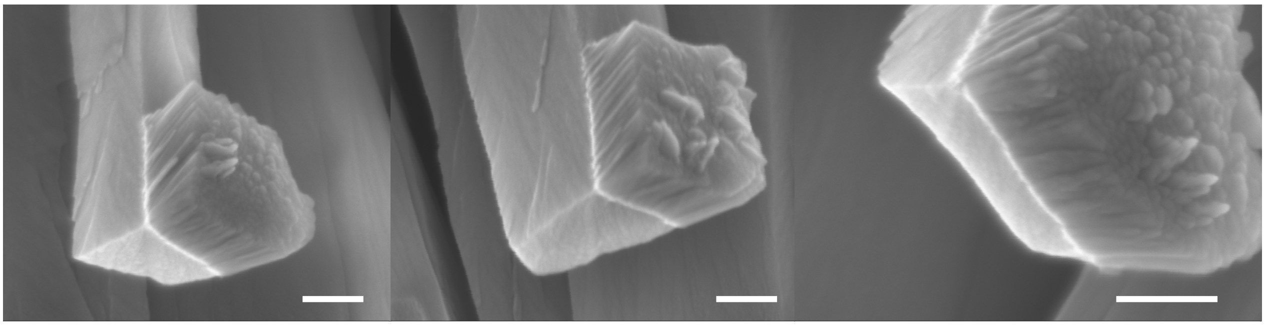

Supplementary Figure 21: SEM micrographs of FeCo-MgO-FeCo trilayer tips with various aspect ratios. Scale bars are 100 nm.

5.6. Perpendicular FeCo film

The morphology of FeCo film deposited onto a perpendicular Si substrate ($\alpha = 0$) at a rate of 3.0 nm/sec is given in Supplementary Figure 22. At thicknesses above 2 μm that is typical for nanowire growth conditions used, perpendicular films show extensive cracking pattern, indicating substantial tensile stress in the film. Surface is rough with height variations up to hundreds of nanometers.

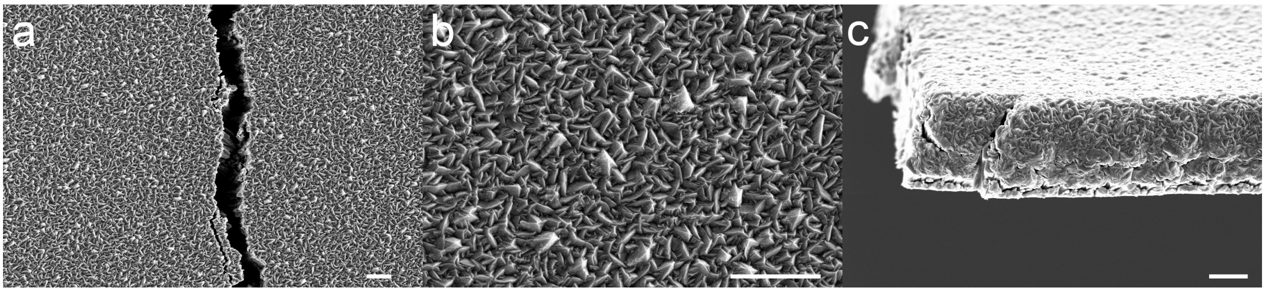

Supplementary Figure 22: SEM examination of a perpendicularly deposited FeCo film (**a**) a crack line in the film. A higher-manification perpendicular view (**b**) and a side view of a delaminated film (**c**). Scale bars are 1 μm.

5.7. Influence of the substrate

We investigated the effect of substrate on the morphology of the resulting FeCo nanowires. We found that Si(100)/Si(111) (native oxide and Si-H terminated), sapphire, glass, kapton tape, and coatings of PMMA and ZEP on flat Si(100) all give indistinguishable results (Supplementary Figure 23). This experiment was conducted at a high evaporation rate of 15 nm/sec.

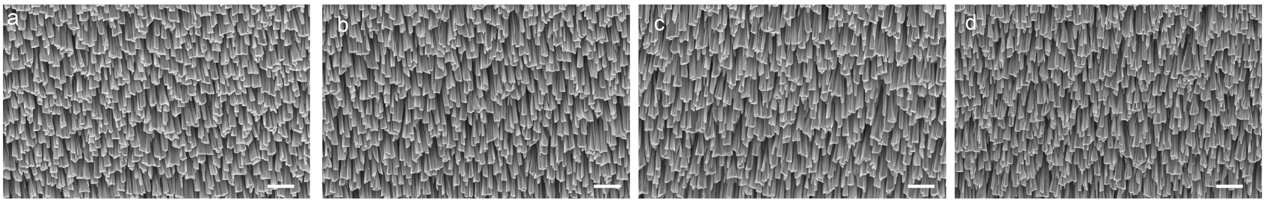

Supplementary Figure 23: Absence of substrate effect. SEM micrographs of FeCo nanowires grown at a deposition rate of 15 nm/sec on (**a**) Si(111), (**b**) sapphire, (**c**) PMMA on Si(100), and (**d**) thermal SiO_2. Scale bars are 1 μm.

5.8. 12 rpm uniform ϕ rotation FeCo nanowires

We have prepared FeCo nanowires under uniform ϕ rotation during growth. Samples were grown with two rotation speeds, 12 rpm and 750 rpm. The experiments show that the rate of rotation, or equivalently the dwell time at each ϕ angle, can be another potent handle for controlling the crystallinity and morphology of GLAD nanowires.

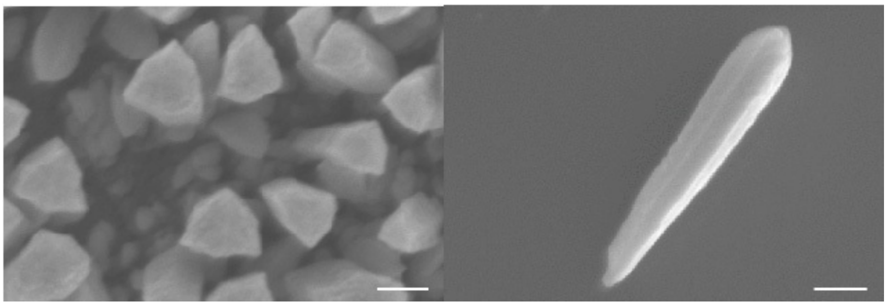

Supplementary Figure 24: SEM micrographs of FeCo nanowires grown with continuous ϕ rotation at 12 rpm. (**a**) a view perpendicular to the substrate. (**b**) A nanowire transferred to a silicon secondary substrate. Scale bars are 100 nm.

We found that nanowires grown at 12 rpm to be single-crystals with wire axes aligned preferentially along the $\langle 1\,1\,1\rangle$ direction (Supplementary Figure 25). The cross sectional profile of the wires is intermediate between equilateral triangles and hexagons (Supplementary Figure 24), reminiscent of the shape of $\langle 1\,1\,1\rangle$ nanowires prepared by stationary toggling-direction deposition (Fig. 3g, 3k in main text). Unlike the latter, however, nanowires grown under uniform ϕ rotation do not show biaxiality, as would be expected due to an isotropic, uniform angular distribution of source vapor.

We further found that smaller crystals that were out-competed during initial nucleation stage were of all possible crystal orientations. This result confirms that the fundamental mechanistic basis for the selection of crystal orientations to be due to be principle of evolutionary selection in competitive growth. A detailed understanding of the underlying atomic processes requires computer simulation work that is beyond the scope of the current study. However, we provide a more detailed discussion in Supplementary Note 1..

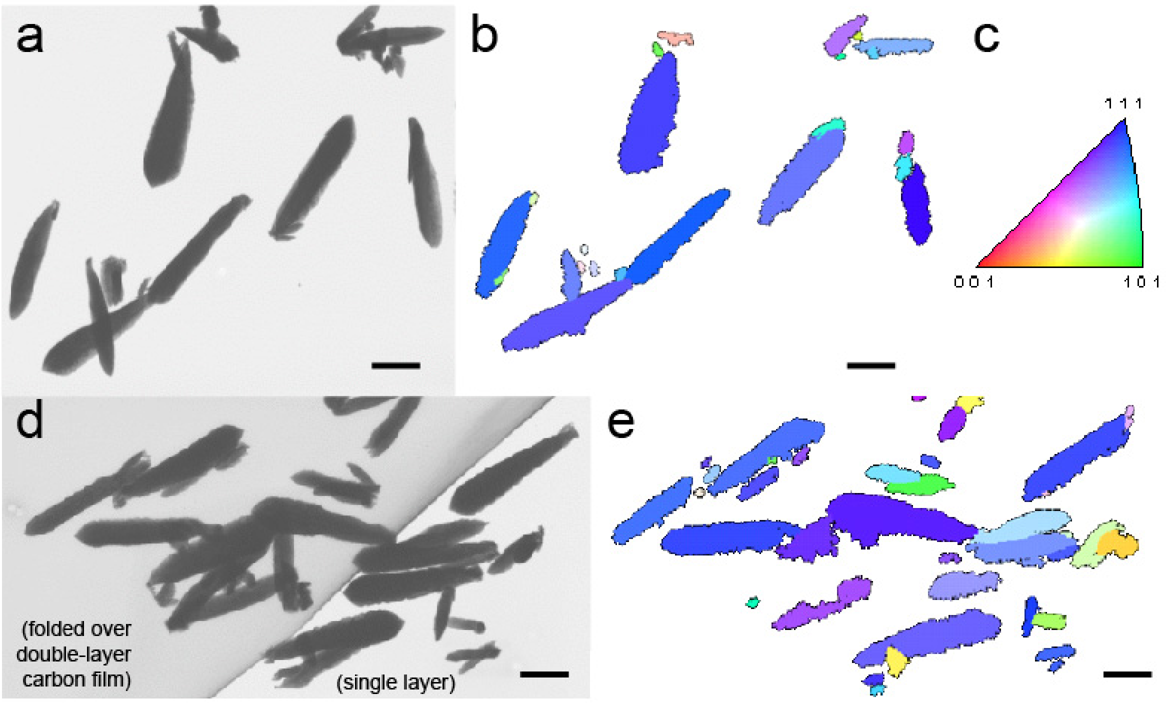

Supplementary Figure 25: Transmission Kikuchi Diffraction (TKD) analysis of FeCo nanowires grown with continuous ϕ rotation at 12 rpm. (**a**) Nanowires strewn on a TEM grid membrane (**b**) TKD data of the region in (**a**). Crystal orientation, encoded in color, has been referenced to the long-axis of each particle. (**c**) Inverse pole figure color legend for datasets in (**b**) and (**e**). (**d**)-(**e**) are another dataset analogous to (**a**) and (**b**). Scale bars are 200 nm.

5.9. 750 rpm uniform ϕ rotation FeCo nanowires

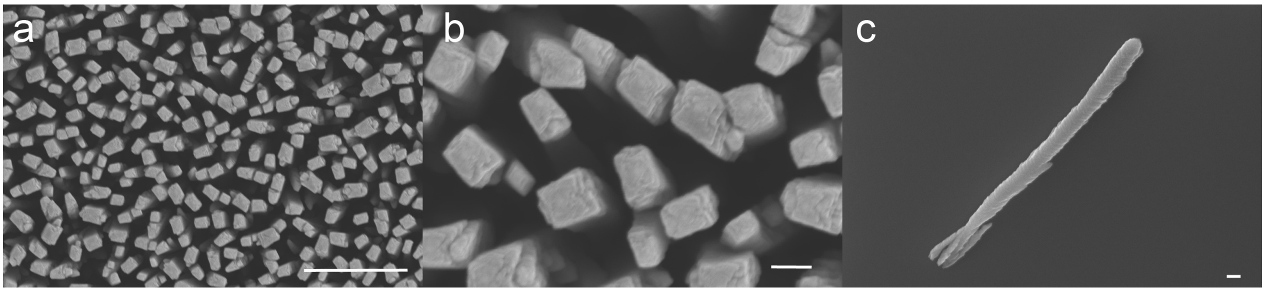

Supplementary Figure 26: SEM micrographs of FeCo nanowires grown with continuous ϕ rotation at 750 rpm. (**a-b**) Views perpendicular to the substrate. (**c**) A nanowire transferred to a silicon secondary substrate. Scale bars are 1 μm in (**a**), and 100 nm in (**b-c**).

Examination of the nanowires grown at 750 rpm shows that the cross sectional profile of the wires is rectangular (Supplementary Figure 26). The orientations of the rectangular cross-sections in the substrate plane is random, as should be expected due to the lack of material flux anisotropy. TKD was attempted on these wires, which show hints of crystallinity, but the diffraction signal was weak

and not compatible with large-area mapping. This is in line with the SEM data that suggest an oriented, polycrystalline overall structure, where the domains are small (Supplementary Figure 26b-c).

6. Additional Hybrid Nanowire Example

6.1. FeCo-silicon hybrid

Silicon is an important material for nanotechnologies and FeCo-silicon hybrid nanowire could have important advantages in certain applications (Supplementary Figure 27). The silicon segment can, for instance, serve as a structural handle to effectively lengthen the nanowire to facilitate micro-manipulation of the magnetic tip. Ferromagnetic-nonmagnetic hybrids are also important building blocks in devices such as on-chip nano rotors [46].

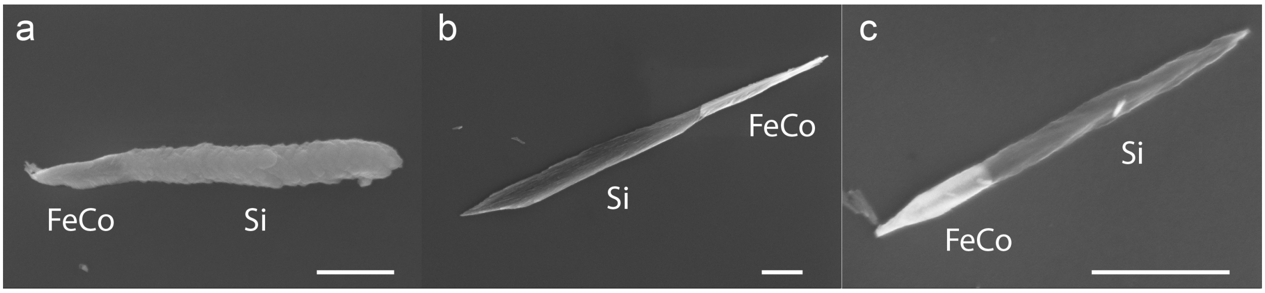

Supplementary Figure 27: SEM micrographs of FeCo-silicon hybrid nanowires deposited on silicon surfaces. (**a**) A sample produced with uniform 12 rpm ϕ rotation throughout both FeCo and silicon depositions. (**b**-**c**) Samples produced without rotation. Scale bars are 500 nm.

7. Dynamic Cantilever Magnetometry

7.1. Saturation magnetization of FeCo nanowires

We have performed dynamic cantilever magnetometry (DCM) on ⟨1 0 0⟩-oriented FeCo nanowires. The principle of the technique [47] and properties of the type of cantilever sensor used [48, 49] have been described. Examples of the data and results are provided in Supplementary Figure 28. The nanowires exhibit high remnance, often up to M_s (Supplementary Figure 28d). We measured saturation magnetization values in the range of 2.0±0.2 T. This value approaches the range for bulk FeCo (2.2-2.4 T).

We believe the slight discrepancy to be caused by impurities and defects incorporated from the gas phase due to insufficient vacuum (10^{-6}-10^{-5} mbar range). See Section 1.7. for additional, complementary discussions. We have performed vibrating-sample magnetometry of FeCo thin film samples prepared under different base pressures and have found that a 256±17 nm film prepared in another system (Evatec in IBM Rueschlikon, Switzerland) with base pressure in the 10^{-8} mbar range to be 2.2±0.2 T, in better agreement with bulk values.

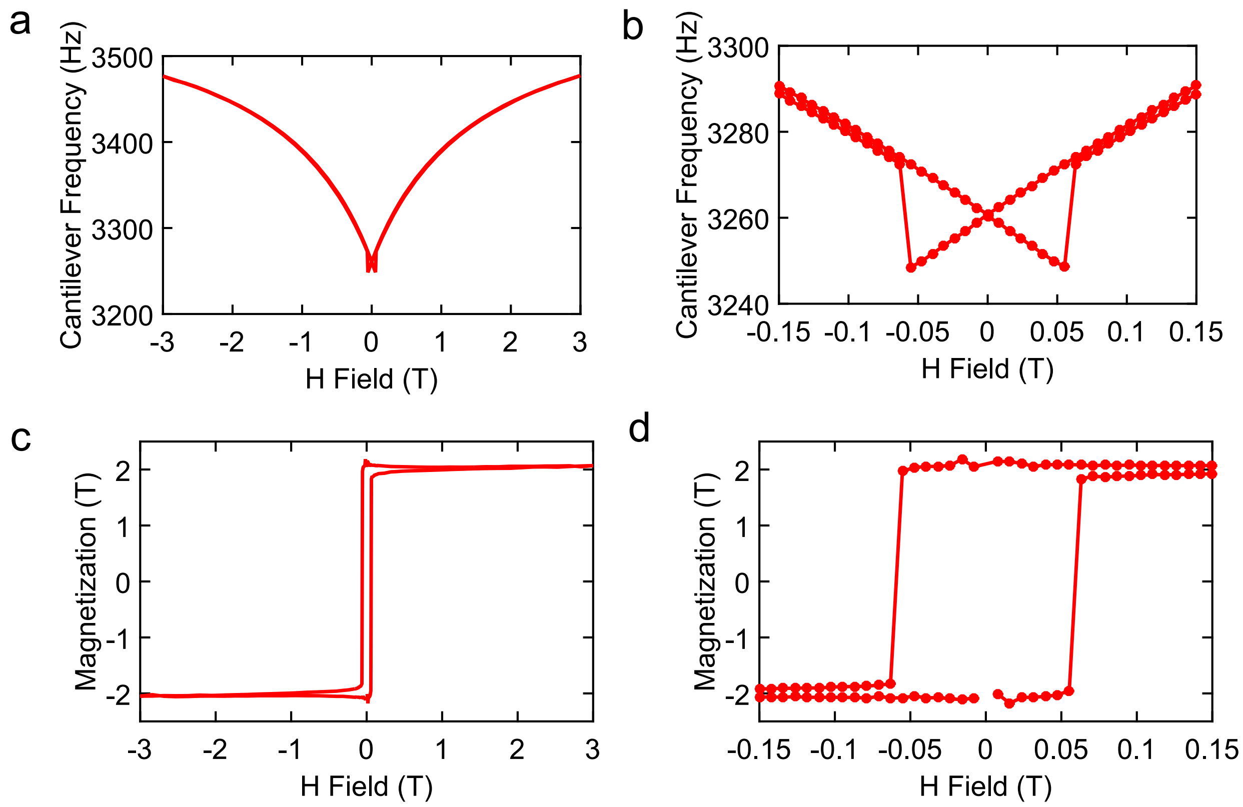

Supplementary Figure 28: Cantilever magnetometry of FeCo nanowires. (**a**) Frequency shift of an silicon cantilever with a FeCo nanowire at the tip (e.g., Supplementary Figure 13) and a zoom-in view (**b**) into the low-field region. (**c-d**) Corresponding magnetization of the nanowire as a function of the H field.

Supplementary References

[1] Zhang, Z. and Lagally, M. G. *Science* **260**, 377–383 (1997).

[2] Evans, J. W. *Phys. Rev. B* **43**, 3897 (1991).

[3] Stroscio, J. A. and Pierce, D. T. *Phys. Rev. B* **49**, 8522(R) (1994).

[4] Hwang, R. Q. and Bartelt, M. C. *Chem. Rev.* **97**, 1063–1082 (1997).

[5] Schröder, J., Günther, C., Hwang, R., and Behm, R. *Ultramicroscopy* **42-44**, 475–482 (1992).

[6] Kopatzki, E., Günther, S., Nichtl-Pecher, W., and Behm, R. *Surf. Sci.* **248**, 154–166 (1993).

[7] Kalff, M., Comsa, G., and Michely, T. *Phys. Rev. Lett.* **81**, 1255 (1998).

[8] Layson, A. R., Evans, J. W., Fournée, V., and Thiel, P. A. *J. Chem. Phys.* **118**, 6467–6472 (2003).

[9] Ling, W. L., Bartelt, N. C., Pohl, K., de la Figuera, J., Hwang, R. Q., and McCarty, K. F. *Phys. Rev. Lett.* **93**, 166101 (2004).

[10] Darling, R. B. EE 527: Microfabrication; Physical Vapor Deposition; Virginia University Lecture, [Online; retrieved December-2016].

[11] Lide, D. R. and Kehiaian, H. V. *CRC Handbook of Thermophysical and Thermochemical Data*. CRC Press, Boca Raton, FL, (1994).

[12] Stuckless, J. T., Frei, N. A., and Campbell, C. T. *Rev. Sci. Instrum.* **69**, 2427–2438 (1998).

[13] Sellers, J. R. V., James, T. E., Hemmingson, S. L., Farmer, J. A., and Campbell, C. T. *Rev. Sci. Instrum.* **84**, 123901 (2013).

[14] Desai, P. D. *J. Phys. Chem. Ref. Data* **15**, 967–983 (1986).

[15] Ou, M. N., Yang, T. J., Harutyunyan, S. R., Chen, Y. Y., Chen, C. D., and Lai, S. J. *Appl. Phys. Lett.* **92**, 063101 (2008).

[16] Völklein, F., Reith, H., Cornelius, T. W., Rauber, M., and Neumann, R. *Nanotechnology* **20**, 325706 (2009).

[17] Yoneoka, S., Lee, J., Liger, M., Yama, G., Kodama, T., Gunji, M., Provine, J., Howe, R. T., Goodson, K. E., and Kenny, T. W. *Nano Lett.* **12**, 683–686 (2012).

[18] Cheng, Z., Liu, L., Xu, S., Lu, M., and Wang, X. *Sci. Rep.* **5**, 10718 (2015).

[19] Lyeo, H.-K. and Cahill, D. G. *Phys. Rev. B* **73**, 144301 (2006).

[20] J. W. Evans, D. E. Sanders, P. A. T. and DePristo, A. E. *Phys. Rev. B* **41**, 5410(R) (1990).

[21] Brune, H., Wintterlin, J., Behm, R. J., and Ertl, G. *Phys. Rev. Lett.* **68**, 624 (1992).

[22] W. F. Egelhoff, J. and Jacob, I. *Phys. Rev. Lett.* **62**, 921 (1989).

[23] Ernst, H.-J., Fabre, F., and Lapujoulade, J. *Surf. Sci. Lett.* **275**, L682–L684 (1992).

[24] Sanders, D. E. and DePristo, A. E. *Surf. Sci.* **254**, 341–353 (1991).

[25] Jensen, K., Kim, K., and Zettl, A. *Nat. Nanotechnol.* **3**, 533–537 (2008).

[26] Chaste, J., Eichler, A., J.Moser, Ceballos, G., Rurali, R., and Bachtold, A. *Nat. Nanotechnol.* **7**, 301–304 (2012).

[27] Hanay, M. S., Kelber, S., Naik, A. K., Chi, D., andE. C. Bullard, S. H., Colinet, E., Duraffourg, L., and Roukes, M. L. *Nat. Nanotechnol.* **7**, 602–608 (2012).

[28] Choi, W. K., Li, L., Chew, H. G., and Zheng, F. *Nanotechnology* **18**, 385302 (2007).

[29] He, Y., Zhao, Y., and Wu, J. *Appl. Phys. Lett.* **92**, 063107 (2008).

[30] Chen, L., Andrea, L., Timalsina, Y. P., Wang, G.-C., and Lu, T.-M. *Cryst. Growth Des.* **13**, 2075–2080 (2013).

[31] Krishnan, R., Riley, M., Lee, S., and Lu, T.-M. *J. Appl. Phys.* **110**, 064311 (2011).

[32] Zhu, H., Cao, W., Larsen, G. K., Toole, R., and Zhao, Y. *J. Vac. Sci. Technol. B* **30**, 030606 (2012).

[33] Patzig, C., Zajadacz, J., Zimmer, K., Fechner, R., Khare, C., and Rauschenbach, B. *Appl. Phys. Lett.* **95**, 103107 (2009).

[34] Ye, D.-X., Karabacak, T., Lim, B. K., Wang, G.-C., and Lu, T.-M. *Nanotechnology* **15**, 817–821 (2004).

[35] Ye, D.-X., Karabacak, T., Picu, R. C., Wang, G.-C., and Lu, T.-M. *Nanotechnology* **16**, 1717–1723 (2005).

[36] Hawkeye, M. M., Taschuk, M. T., and Brett, M. J. *Glancing Angle Deposition of Thin Films: Engineering the Nanoscale.* John-Wiley & Sons, Ltd, (2014).

[37] Zhou, L. G. and Huang, H. *Phys. Rev. Lett.* **101**, 266102 (2008).

[38] Shu, D.-J., Xiong, X., Wang, Z.-W., Zhang, Z., Wang, M., and Ming, N.-B. *J. Phys. Chem. C* **115**, 31–36 (2011).

[39] Patzig, C. and Rauschenbach, B. *J. Vac. Sci. Technol. B* **25**, 833 (2007).

[40] Elliott, P. R., Stagon, S. P., and Huang, H. *Sci. Rep.* **5**, 16826 (2015).

[41] Asano, T., Uetake, N., and Suzuki, K. *J. Nucl. Sci. Technol.* **29**, 1194–1200 (1992).

[42] Adams, B. L., Wright, S. I., and Kunze, K. *Metall. Trans. A* **24**, 819–831 (1993).

[43] Zaefferer, S. *Ultramicroscopy* **107**, 254–266 (2007).

[44] Keller, R. R. and Geiss, R. H. *J. Microsc.* **245**, 245–251 (2012).

[45] Trimby, P. W. *Ultramicroscopy* **120**, 16–24 (2012).

[46] Kim, K., Xu, X., Guo, J., and Fan, D. L. *Nat. Commun.* **5:3632** (2014).

[47] Stipe, B. C., Mamin, H. J., Stowe, T. D., Kenny, T. W., and Rugar, D. *Phys. Rev. Lett.* **86**,

2874 (2001).

[48] Longenecker, J. G., Mamin, H. J., Senko, A. W., Chen, L., Rettner, C. T., Rugar, D., and Marohn, J. A. *ACS Nano* **6**, 9637–9645 (2012).

[49] Weber, D. P., Ruffer, D., Buchter, A., Xue, F., Russo-Averchi, E., Huber, R., Berberich, P., Arbiol, J., i Morral, A. F., Grundler, D., and Poggio, M. *Nano Lett.* **12**, 6139–6144 (2012).
